# Association of Tat with Promoters of PTEN and PP2A Subunits Is Key to Transcriptional Activation of Apoptotic Pathways in HIV-Infected CD4+ T Cells

**DOI:** 10.1371/journal.ppat.1001103

**Published:** 2010-09-16

**Authors:** Nayoung Kim, Sami Kukkonen, Sumeet Gupta, Anna Aldovini

**Affiliations:** 1 Children's Hospital Boston, Department of Medicine, and Harvard Medical School, Department of Pediatrics, Boston, Massachusetts, United States of America; 2 Whitehead Institute for Biomedical Research, Cambridge, Massachusetts, United States of America; Baylor College of Medicine, United States of America

## Abstract

Apoptosis in HIV-1-infected CD4+ primary T cells is triggered by the alteration of the PI3K and p53 pathways, which converge on the FOXO3a transcriptional activator. Tat alone can cause activation of FOXO3a and of its proapoptotic target genes. To understand how Tat affects this pathway, we carried out ChIP-Chip experiments with Tat. Tat associates with the promoters of PTEN and two PP2A subunit genes, but not with the FOXO3a promoter. PTEN and PP2A encode phosphatases, whose levels and activity are increased when Tat is expressed. They counteract phosphorylation of Akt1 and FOXO3a, and so activate transcriptional activity of FOXO3a. FOXO3a promotes increased transcription of Egr-1, which can further stimulate the transcription of PTEN, thereby reinforcing the pathway that leads to FOXO3a transcriptional activation. RNAi experiments support the role of PTEN and PP2A in the initiation of the Tat-mediated cascade, which is critical to apoptosis. The increased accumulation of PTEN and PP2A subunit mRNAs during Tat expression is more likely to be the result of increased transcription initiation and not relief of promoter-proximal pausing of RNAPII. The Tat-PTEN and -PP2A promoter interactions provide a mechanistic explanation of Tat-mediated apoptosis in CD4+ T cells.

## Author Summary

HIV infection leads to the depletion of CD4+ T cells, the major viral cell target. The destruction of these cells can occur because of cytopathic effect or apoptosis. HIV Tat is one of the proteins that can contribute to the apoptotic process of both infected and uninfected cells, as it is released in the plasma and enter uninfected cells. Tat expression in CD4+ T-cells is linked to increased transcriptional activity of FOXO3a, a factor that targets the transcription of pro-apoptotic genes. The mechanism by which Tat leads to activation apoptotic pathways is by associating with the promoters of the phospatases PTEN and PP2A and by increasing their levels. The increased amount of these proteins leads to a decreased amount of pAKt1 and increased amount of non-phosphorylated FOXO3a, which migrates from the cytoplasm to the nucleus and increases the transcription of its proapoptotic target genes. These results, together with experiments that silence PTEN and PP2A and measure their activities, identify the association of Tat with PTEN and PP2A promoters as the initiating event of Tat-mediated apoptosis.

## Introduction

HIV-1-infected CD4+ primary T cells progress to the G0 phase of the cell cycle and to cell death [1]. Apoptosis in these cells is triggered by the alteration of transcriptional pathways that converge on the Forkhead box O3 (FOXO3a) transcriptional activator. The induction of FOXO3a target genes, such as Bcl-2-like 11 (BCL2L11 or Bim), TNF-related apoptosis-inducing ligand (TRAIL) and Fas ligand (FasL or CD95L), activates apoptotic intrinsic (via Bim) and extrinsic pathways [2], [3], indicating that HIV infection leads to apoptosis by the engagement of multiple apoptotic pathways. The induction of phosphatase and tensin homolog (PTEN) and FOXO3a was observed in cells that express only the Tat protein, suggesting that Tat may be a key player in the activation of these pathways.

PTEN reduces the phosphorylation of Akt1 and expression of PTEN is transcriptionally regulated by the Early Growth Response Protein 1 (Egr-1) [4], [5], [6]. Egr-1 is expressed at higher levels in HIV-infected T cells [1]. Increased expression of PTEN reduces serine/threonine protein kinase pAkt1 levels, which cause reduced phosphorylation of FOXO3a. Unphosphorylated FOXO3a translocates to the nucleus and becomes transcriptionally active [7].

Transcription of HIV genes from the HIV long terminal repeat (LTR) is strictly dependent on Tat, which interacts with the Positive Transcription Elongation Factor b (P-TEFb) and histone acetyltransferases [8]. The interaction with P-TEFb occurs at the trans-activation-responsive (TAR) element of the nascent RNA and mediates the relief of RNA polymerase II (RNAPII) pausing that occurs at TAR. Tat transcriptional activity is also dependent on lysine acetylation mediated by nuclear histone acetyltransferases p300/CBP (E1A binding protein p300/CREB binding protein) and PCAF (P300/CBP-associated factor). The p300/CBP complex is a transcriptional coactivator of Egr-1 [9], [10], [11], [12]. Tat may enhance the transcriptional activity of p300/CBP by increasing the histone acetyl transferase (HAT) activity on the PTEN promoter, as for histone H4 and the HIV LTR [13]. Inhibition of Sirtuin 1 (SIRT1) deacetylase activity by Tat [14], might also increase transcription of PTEN. Tat can be found in patients' serum [15], [16] and can cross the cell membrane to enter cells [17]. Tat could thus play a role in the apoptosis of uninfected cells by activating the PTEN-FOXO3a pathway after entry. The survival of memory CD4+ T cells correlates with the phosphorylated levels of FOXO3a. The levels of phospho-FOXO3a are reduced in HIV-infected individuals and are higher in elite controllers, who control viral replication to undetectable viremia in the absence of therapy [18], [19]. Activation of the PTEN-FOXO3a pathway via the Tat protein could be the mechanism by which apoptosis is triggered in HIV- infected and non-infected cells and explain the significant decline of the CD4+ T cell memory population in HIV-1-infected individuals [1].

Here we show that the Tat protein triggers apoptosis by altering the Akt-FOXO3a-Egr-1 pathway via its interaction with the promoters of two phosphatases, PTEN and Protein phosphatase 2 (PP2A).

## Results

### Tat-mediated cellular modulation of gene expression in Jurkat T cells

**Figure 1:**
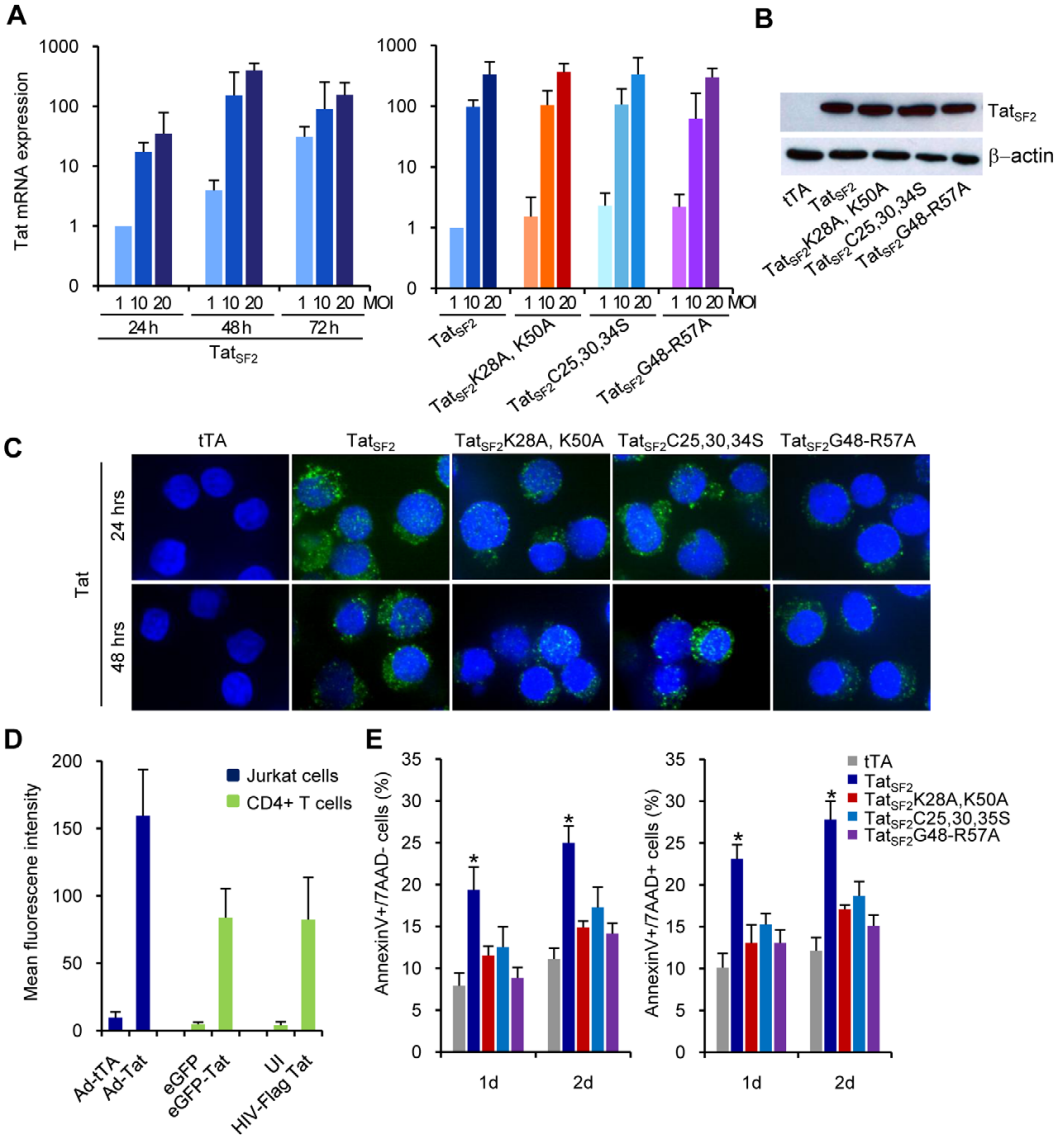
Tat and Tat mutants expression and apoptosis in Jurkat T cells. A. mRNA levels of Tat in Jurkat T cells expressing Tat_SF2_ at 24, 48, and 72 hrs after infection at MOI of 1, 10, and 20 (first panel) or wt and Tat mutants at 48 hrs after infection at MOI of 1, 10, and 20 (second panel), analyzed by qRT-PCR. Results are normalized to GAPDH and reported as fold induction relative to Ad-Tat samples infected at MOI of 1. The means ± SEM derived from three independent experiments are reported. B. Western blot analysis of Tat expression in Jurkat cells. C. Detection of wild type and mutant Tat in Jurkat cells. Nuclei are counterstained with DRAG5. D. Tat protein mean fluorescence intensity (MFI) of three independent flow cytometric analyses of Jurkat cells, after infection with different Tat expressing viruses. E. Apoptosis in Tat-expressing Jurkat T cells. Levels of early and late apoptosis are reported as percentage of Jurkat T cells that stain for Annexin V only (left panel) or Annexin V and 7AAD (right panel). The means ± SEM of three experiments are shown. *, *p*<0.05 when Tat_SF2_ is compared to tTA control.

We reported that HIV-1 Tat-induced FOXO3a is a key mediator of apoptosis in HIV-1-infected primary human CD4 T lymphocytes [1]. To gain insight into the molecular mechanism by which Tat protein affects the PTEN-FOXO3a-Egr-1 signaling pathway, we investigated the impact of the HIV-1 Tat protein on the regulation of FOXO3a in Jurkat T cells. Jurkat T cells are susceptible to adenovirus infection due to high surface levels of the Coxsackie adenovirus receptor (CAR) [20]. Because retroviral transduction of primary CD4+ lymphocytes is inefficient, the use of adenovirus-mediated *tat* gene transfer in Jurkat T cells is a more amenable model for mechanistic studies. We investigated whether Tat expression in Jurkat cells resulted in modulatory effects on expression of proapoptotic genes, similar to those we observed in primary CD4+ T cells infected with HIVΔ2GFP, HIVΔ3GFP, and wild-type viruses [1]. Jurkat T cells were infected with an Ad-Tat vector expressing Tat in combination with the transactivator Ad-tTA, required to induce Tat expression, or Ad-tTA alone as a control. We conducted real-time RT-PCR using RNA obtained from Jurkat T cells infected with Ad-Tat_SF2_ (a 101 amino acid wild type Tat protein from HIV_SF2_), Ad-Tat_SF2_K28A,K50A, a mutant that does not associate with p300 [21]–[22]), Ad-Tat_SF2_C25,30,35S, a mutant that does not interact with P-TEFb [23], and Ad-Tat_SF2_G48-R57A, a mutant lacking the nuclear localization signal (NLS), in which the key residues of the NLS are substituted by alanines [24]. RNA was isolated from infected cells 24 48, and 72 hours post-infection at different MOI. Real-time RT-PCR was carried out with primers for glyceraldehyde-3-phosphate dehydrogenase (GAPDH) for normalization. Tat mRNA expression and Tat protein level accumulation was comparable for all cultures examined (Figure 1A and 1B). Intracellular staining for Tat on samples obtained 24 and 48 hrs after Ad-Tat_SF2_ infection ranged between 40 and 60 percent positive cells (data not shown) and was found both in the cytoplasm and in the nucleus in the case of the wild type and the mutants excepts for Tat_SF2_G48-R57A, with was detected virtually exclusively in the cytoplasm (Figure 1C). Levels of Tat expression obtained with different vectors were evaluated by flow cytometry. The Mean Fluorescence Intensity (MFI) for Tat expression after infection with Ad-Tat, eGFP-Tat (a retroviral vector expressing Tat) and HIV-Flag Tat (a HIV infectious virus in which Tat has been tagged with the FLAG epitope) is reported in Figure 1D. Tat MFI was approximately the same after retroviral expression vector or HIV infection and two-fold higher after Ad-Tat_SF2_ infection (Figure 1D). Rates of early and late apoptosis were measured by evaluating the number of Annexin V+/7AAD- and Annexin V+/7AAD+ cells. A significant (p<0.05) increase in apoptosis was observed in Jurkat cells infected with Ad-Tat_SF2_ (Figure 1E) compared to control or Tat mutants.

**Figure 2:**
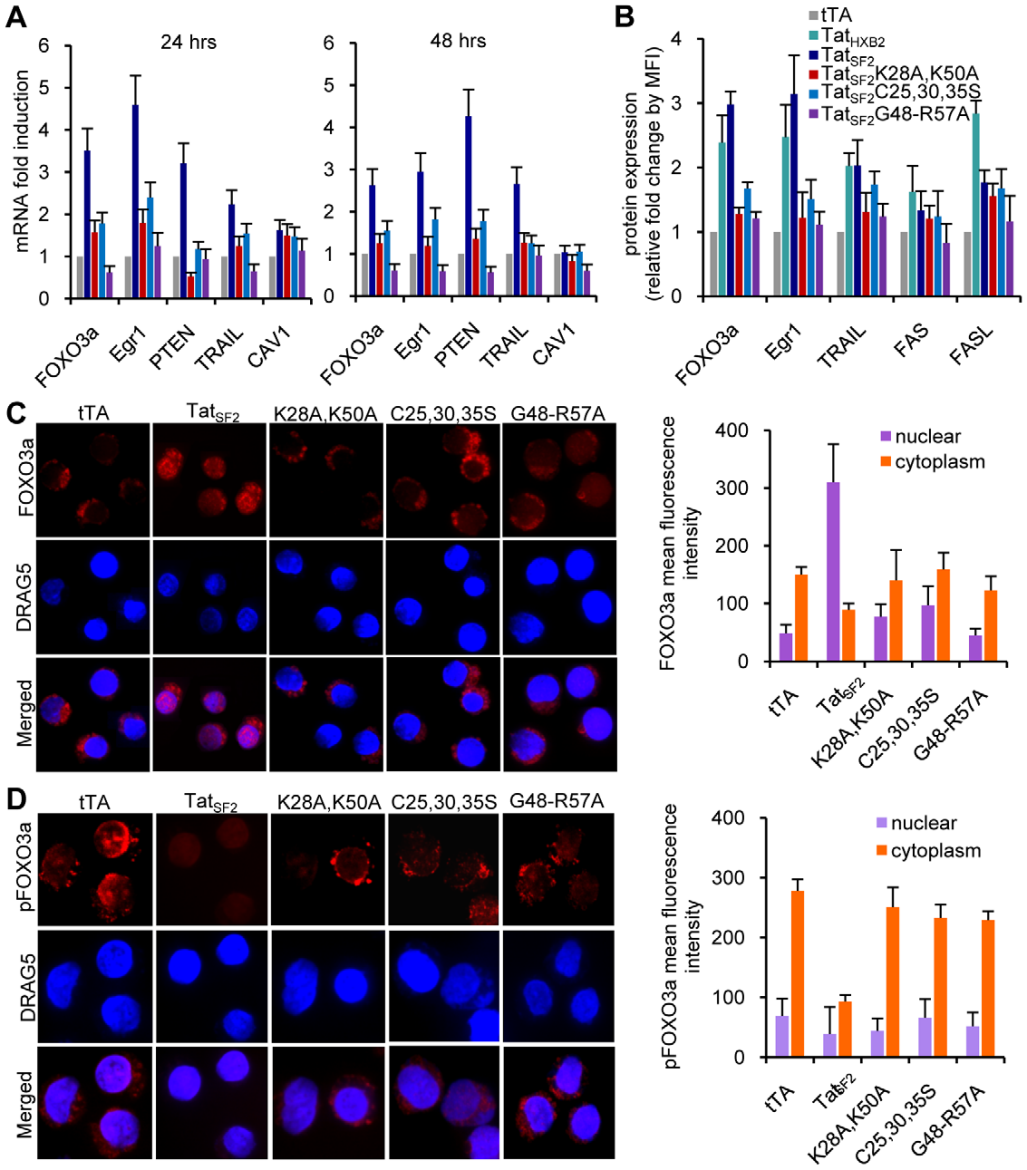
Tat-mediated cellular gene modulation in Jurkat T cells. (A) mRNA levels of selected cellular genes 24 and 48 hours after infection with adenoviral vectors expressing wild type Tat and Tat mutants. B. Protein levels of cellular genes analyzed by flow cytometry analysis. Results are reported as fold increase of mean fluorescence intensity (MFI) relative to the tTA control. C. HIV-1 Tat increases FOXO3a nuclear localization in Jurkat T cells. Jurkat T cells infected with Ad-tTA or Ad-tTA+Ad-Tat_SF2_ were analyzed by confocal microscopy 24 hrs after infection. Cells were stained with DRAG5 to visualize the nucleus (blue), and FOXO3a cellular localization (red) was detected using antibodies against FOXO3a (C) or p-FOXO3a (D). A quantitative analysis of nuclear and cytoplasmic FOXO3a or p-FOXO3a fluorescence intensity is provided at the right of each panel.

We found that genes up-regulated in primary CD4+ T cells infected with HIVΔ2GFP and HIVΔ3GFP or in HeLa cells infected with Ad-Tat [1] were similarly modulated in Jurkat T cells infected with Ad-Tat_SF2_ (Figure 2A). The Tat mutants had substantially less effect on the expression of these genes. FOXO3a, Egr-1, and PTEN, and TRAIL, critical to apoptosis in primary CD4+ T cells [1], were also induced by Tat expression in Jurkat cells. We found increased accumulation of FOXO3a, Egr-1, and TRAIL as assessed cytofluorimetrically (Figure 2B). The pattern of protein expression was similar when Jurkat cells were infected with adenoviruses expressing two different Tat alleles, Tat_SF2_ and Tat_HXB2_ (a 86 amino acid Tat protein, which misses 15 residues at the carboxyl-terminus) and was not significantly altered after infection with the mutants. The subcellular localization of FOXO3a was assessed by immunofluorescence microscopy using antibodies against FOXO3a and pFOXO3a (Figure 2C and 2D). In the Ad-tTA control, the majority of FOXO3a was phosphorylated (inactive form) and distributed in the cytoplasm (Figure 2D, first column). In contrast, there was significantly less cytoplasmic pFOXO3a in cells infected with Ad-Tat_SF2_ (Figure 2D, second column). We observed different results using anti-FOXO3a antibody. FOXO3a was mostly in the cytoplasm when cells were infected with Ad-tTA alone (Figure 2C, first column). However, Tat expression increased the amount and the translocation of FOXO3a from the cytoplasm to the nucleus (active form), indicating that FOXO3a was no longer phosphorylated (Figure 2C, second column). The intensity of nuclear FOXO fluorescence was higher in cells infected with Ad-Tat_SF2_ than in those infected with Ad-tTA alone. Cells infected with the Tat mutants showed a patter similar to those infected with Ad-tTA. HIV-1 Tat thus increases intracellular levels of FOXO3a, as suggested by RT-PCR for the FOXO3a RNA.

These data confirm the role of Tat in the activation of FOXO3a and in the induction of its target genes involved in apoptosis in Jurkat cells and validate its use to investigate the mechanisms by which HIV-1 Tat affects the PTEN-FOXO3a pathway in primary CD4+ T cells.

### Tat associates with the PPP2R1B and PPP2R5E promoters to increase PPP2R1B and PPP2R5E RNA and protein levels as well as PP2A activity in Jurkat cells

**Figure 3:**
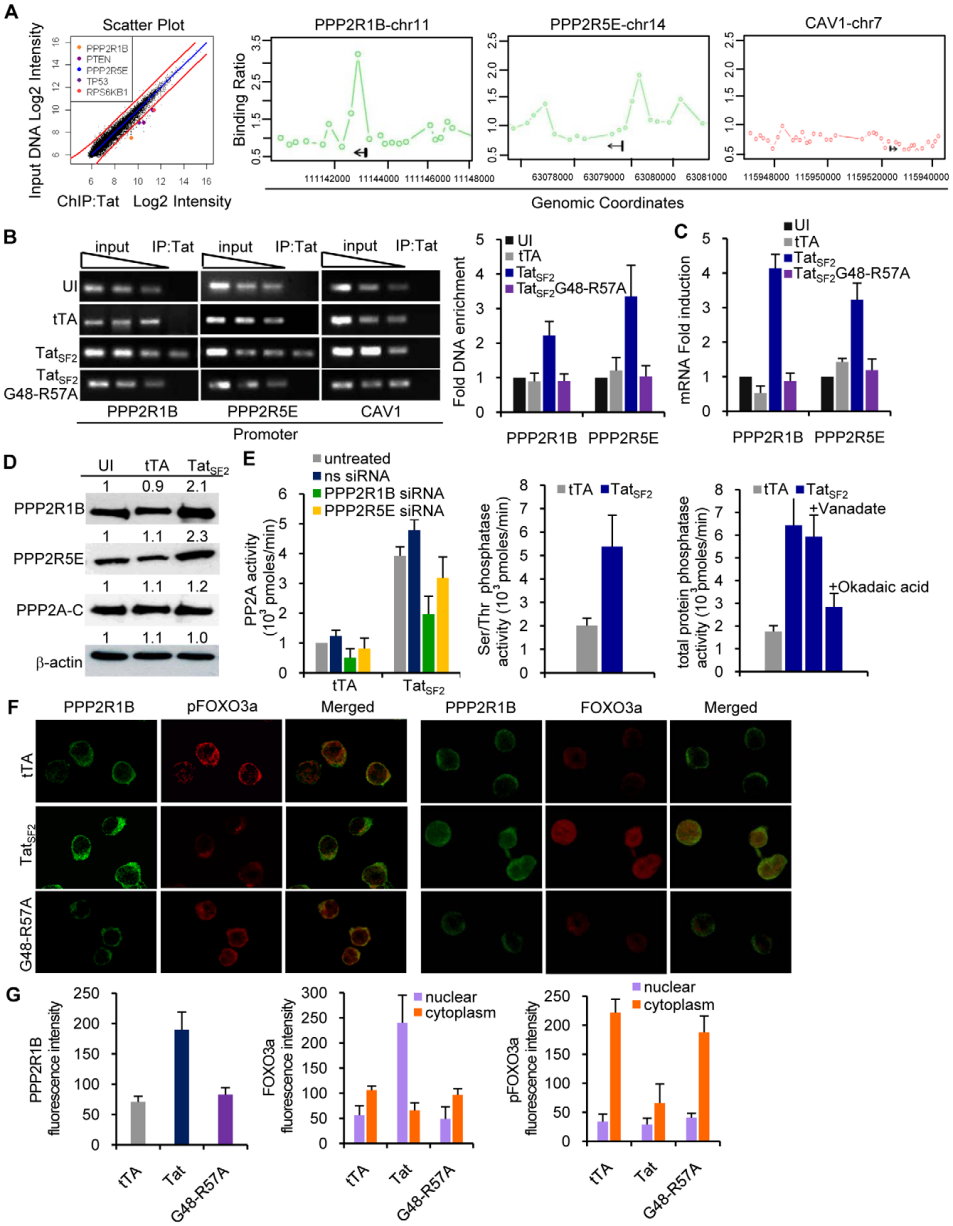
Tat associates with the PPP2R1B and PPP2R5E promoter and increases protein levels of PPP2R1B and PPP2R5E and PP2A activity in Jurkat cells. **A.** Genes enriched in the Tat-immunoprecipitated DNA and associated with the PI3K pathway show a hybridization intensity with a *P* value lower than 0.001 (hybridization intensities with higher P values fall within the red lines). PPP2R1B, PPP2R5E, and Caveolin 1 (CAV1, negative control) promoter enrichment ratio (ChIP versus total input DNA in ChIP-on-Chip analysis) in Jurkat cells expressing Tat_SF2_. **B.** ChIP analysis of the PPP2R1B and PPP2R5E promoters in Jurkat T cells expressing Tat_SF2_. DNA from input (90, 30, 10 ng of DNA) and immunoprecipitated samples (3 ng of DNA) was amplified by standard PCR (P2 set of primers, see Supplemental Table S2) and run on 2% agarose gel (second and third panels). One representative experiment is shown in the 3 left panels. In the right panel, the average fold enrichment of a certain promoter in the immunoprecipitated DNA relative to input DNA ± SEM from three independent qPCR experiments is reported. All cycle threshold (Ct) values obtained with 10 ng of immunoprecipitated DNAs were compared with the Ct value obtained with 10 ng of the corresponding input DNA. **C.** mRNA levels of PPP2R1B and PPP2R5E in Jurkat T cells expressing Tat_SF2_ or the mutant Tat_SF2_G48-R57A, analyzed by qRT-PCR. Results are normalized to GAPDH and reported as fold induction relative to uninfected samples. The means ± SEM of three experiments are shown. **D.** Western blot analysis of PPP2R1B, PPP2R5E, and the catalytic subunit PP2A-C. Fold-increase compared to the uninfected control (UI) is indicated above the band. **E.** PP2A enzyme activity in lysates from Jurkat cells expressing Tat_SF2_ alone or in the presence of siRNAs (left panel); serine/threonine phosphatase activity (middle panel), and total phosphatase activity (right panel) in lysates from Jurkat cells infected with Adeno-Tat_SF2_ or with the Adeno-tTA control. Inhibition of tyrosine phosphatases and serine/threonine phosphatases was carried out by incubation of the lysate with sodium vanadate (1 mM) or okadaic acid (0.25 mM) in the phosphatase assay buffer. **F.** Tat increases PP2A expression and FOXO3a nuclear localization in Jurkat T cells. Jurkat T cells expressing tTA alone, Tat_SF2_+tTA, or Tat_SF2_G48-R57A +tTA were stained with antibodies against PPP2R1B (first and forth columns of panels, green), pFOXO3a (second column, red), and FOXO3a (forth column, red) and analyzed by confocal microscopy. Merged images are shown in the panels in the third and sixth columns. **G.** Quantitative analysis of the fluorescence intensity of cytoplasmic PPP2R1B, and of cytoplasmic and nuclear FOXO3a and pFOXO3a in Jurkat T cells expressing or tTA alone, Tat_SF2_+tTA, or Tat_SF2_G48-R57A +tTA. PPP2R1B and FOXO3a fluorescence was expressed as total intensity per cell (pixels above threshold x fluorescence intensity). Bars indicate the mean ± SEM of triplicate assays from two separate experiments. At least 100 cells were counted for each condition.

How does Tat modulate cellular gene expression during HIV-1 mediated apoptosis? We used ChIP coupled with promoter DNA microarray analysis (ChIP-Chip) [25] to identify genes whose promoters may associate with Tat. We infected Jurkat cells for 6 hours with an adenovirus expressing FLAG-tagged Tat_SF2_ or the mutant Ad-Tat_SF2_G48-R57A lacking the NLS as a negative control. An anti-FLAG antibody suitable for use in ChIP experiments was used because of its lack of background [26], [27]. Two independent experiments were analyzed. We selected a stringent P value threshold of 0.001 to identify genes bound by Tat (Figure 3A). In cells expressing Tat_SF2_, we identified 450 promoters that were occupied by Tat (*P*<0.001). Control experiments in cells expressing the negative control Tat_SF2_G48-R57A showed only 12 positive signals, which is an acceptable background for ChIP-Chip experiments, supporting the specificity of the results observed with Ad-Tat_SF2_. The 450 genes whose promoters associate with Tat_SF2_ encode proteins that affect many cellular processes, including transcriptional regulation, apoptosis, cell cycle, and immune response. They are listed in Supplemental Table S1, with the *P* value and the fold enrichment of their association to the promoter compared to input DNA.

To identify the cellular functions affected by the Tat protein, we compared the list of genes occupied by Tat with the biological pathways annotated by the Ingenuity Pathway Analysis (IPA). We found that Tat target genes are significantly associated with promoters of genes that are part of a few pathways (Table 1). A subset of these genes belongs to the PI3K signaling pathway (p = 3.39E-03) (Figure 3A). Among these genes were PTEN, a gene that encodes a truncated, non-functional phosphatase in Jurkat cells [28], [29], and PPP2R1B and PPP2R5E, regulatory subunits of protein phosphatase 2A (PP2A). PPP2R1B (PR65β) is a regulatory subunit A β isoform, tightly associated with the PP2A catalytic subunit C, to form a scaffold onto which the appropriate B subunit can bind. PPP2R5E (B56ε) is a member of the B56 regulatory subunit ε isoform involved in multiple signaling pathways [30], [31], [32], [33], [34], [35], [36], [37]. PP2A affects the phosphorylation status of Akt1 and FOXO3a [38], [39], [40], [41], [42]. High levels of PP2A correlate with reduced phosphorylation of FOXO3a and consequently increase its transcriptional activity [43], [44]. The ChIP-Chip results of Tat binding to the promoters of PPP2R1B and PPP2R5E were further validated by conventional ChIP, performed using site-specific primers on chromatin precipitated from uninfected cells and cells expressing Tat_SF2_ or tTA alone. Primers were designed near the site represented by the oligonucleotides on the promoter arrays that provided a positive signal and were used for qPCR amplification of the corresponding sequences present in the immunoprecipitation-captured chromatin. When the immunoprecipitated DNA samples were evaluated by qPCR, PPP2R1B and PPP2R5E promoter sequences were enriched in IP-captured chromatin from cells expressing Tat_SF2_ compared to the control cells (Figure 3B). To determine whether association of Tat with the PPP2R1B and PPP2R5E promoters affects gene expression, we carried out qRT-PCR with RNA from Tat-expressing cells. mRNA levels of PPP2R1B and PPP2R5E were elevated in cells that express Tat_SF2_, compared to untreated cells or cells that express tTA alone (Figure 3C).

**Table 1:** Analysis of promoters found associated with HIV-Tat in jurkat cells.

Ingenuity Signaling Pathway	p-Value
Hormone Receptor Regulated Cholesterol Metabolism	2.36E-03
PI3K Signaling*	3.39E-03
G2/M Transition of the Cell Cycle	2.05E-02
Mitochondrial Dysfunction	2.92E-02
PPARa/RXR Activation	3.26E-02

(*) PI3K pathway related genes bound by HIV-1 Tat	
Gene	Description
PPP2R1B	protein phosphatase 2, regulatory subunit A, beta isoform
PPP2R5E	protein phosphatase 2, regulatory subunit B', epsilon isoform
PTEN	phosphatase and tensin homolog
RPS6KB1	ribosomal protein S6 kinase, 70 kDa, polypeptide 1
p53	tumor protein p53

These results confirmed the association of Tat with the PPP2R1B and PPP2R5E promoters identified by the ChIP-Chip analysis and support a role for this association in increased gene expression of PPP2R1B and PPP2R5E seen in Tat expressing Jurkat cells.

We further evaluated the effects of Tat on the protein levels of PPP2R1B and PPP2R5E by immunoblot. PP2A is a ubiquitous enzyme with pleiotropic functions. PP2A is a heterotrimer that consists of a catalytic C subunit, a regulatory A subunit, and a variable regulatory B subunit. Regulation is accomplished mainly by members of a family of regulatory subunits, which determine the substrate specificity. The protein content of PP2A-C was not altered in cells infected with Ad-Tat_SF2_ or Ad-tTA (Figure 3D). In contrast, an approximately two-fold increase in the amount of PPP2R1B and PPP2R5E protein was observed in cells infected with Ad-Tat_SF2_ (Figure 3D).

Does the increase in PPP2R1B and PPP2R5E protein levels correlate with increased PP2A activity and serine/threonine phosphatase activity? We carried out an immunocomplex protein phosphatase assay and a malachite green assay on lysates from cells infected with Ad-Tat_SF2_ or Ad-tTA alone, and treated with a non specific siRNA, a PPP2R1B siRNA, or a PPP2R5E siRNA (Figure 3E, left panel). Cells that express Tat_SF2_ showed an approximately 2-fold increase in PP2A activity compared to cells that were infected with Ad-tTA only or were treated with PP2A subunit specific siRNAs, and a similar two-fold increase in total serine/threonine phosphatase activity (Figure 3E, middle panel), linking increased PP2A activity to the increase in PPP2R1B and PPP2R5E. When we measured total phosphatase activity, we found an approximately 4-fold increase in cells infected with Ad-Tat_SF2_+Ad-tTA compared to the Ad-tTA alone control. Total phosphatase activity in cells that express Tat was reduced to approximately two-fold when cells were treated with okadaic acid, an inhibitor of PP2A, but not when treated with sodium vanadate, a generic inhibitor of tyrosine phosphatases (Figure 3E, right panel). The increase in PPP2R1B and PPP2R5E protein induced by Tat is therefore the critical determinant of the increased serine/threonine phosphatase activity observed in Tat-expressing cells.

We examined the cellular localization of PP2A and FOXO3a in the presence or absence of Tat. Jurkat T cells were infected for 24 hours with Ad-Tat_SF2_ + Ad-tTA or Ad-tTA alone. The subcellular localization of FOXO3a and PPP2R1B was assessed by immunofluorescence microscopy using antibodies against pFOXO3a, FOXO3a, and PPP2R1B (Figure 3F). Cells infected with Ad-Tat_SF2_ revealed stronger fluorescence intensity of PPP2R1B compared to those infected with Ad-tTA alone. This observation confirmed the effect of Tat on the PPP2R1B protein levels detected by Western blot analysis (Figure 3D). Tat expression was associated with increased amounts of nuclear FOXO3a (Figure 3F and 3G). In contrast, FOXO3a was detected predominantly in the cytoplasm in cells infected with Ad-tTA alone or the Tat mutant (Figure 3F, 3G). These observations further confirm that HIV-1 Tat increases PP2A expression and FOXO3a nuclear translocation.

### siRNA-mediated PP2A knockdown reduces Tat-induced apoptosis in Jurkat cells

**Figure 4:**
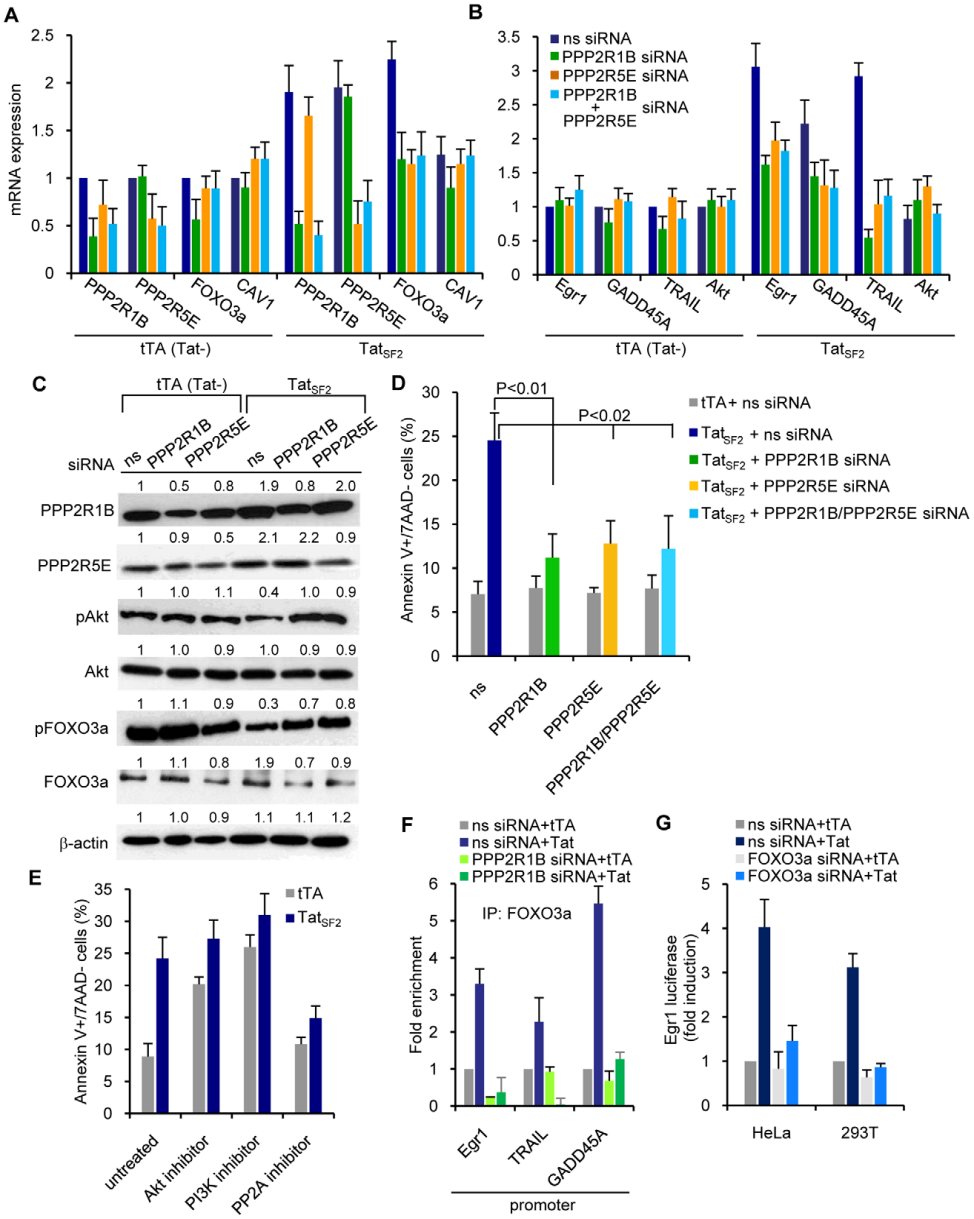
siRNA-mediated knockdown of PP2A reduces Tat-induced apoptosis in Jurkat T cells. mRNA expression levels of (**A**) PPP2R1B and PPP2R5E and FOXO3a and (**B**) Egr1, GADD45A, and TRAIL [1] in Jurkat T cells expressing Tat_SF2_+tTA or tTA alone. Results are normalized to GAPDH and reported as fold induction relative to tTA expressing cells treated with ns siRNA. **C.** Western blot analysis of cell lysates treated with PPP2R1B and/or PPP2R5E siRNA or ns siRNA. **D.** Levels of apoptosis in the same cells 48 h after siRNA transduction, measured by staining for Annexin V and 7AAD. The means ± SEM of three independent experiments are reported. **E.** Levels of apoptosis in Jurkat cells infected with Ad-tTA or Ad-Tat and treated with an Akt inhibitor (50 µM Akt1-1/2), a PI3K inhibitor (10 nM LY294002), or a PP2A inhibitor (100 nM okadaic acid), measured by staining for Annexin V and 7AAD. **F.** ChIP analysis of the promoters of three FOXO3a target genes. ChIP was carried out with an anti-FOXO3a antibody in lysates of Jurkat T cells treated with ns siRNA or PPP2R1B siRNA and expressing Tat_SF2_ or Tat_SF2_K28A,K50A. Recovered DNA was analyzed by qPCR using primers specific for the Egr1, TRAIL, and GADD45A promoters. The average fold enrichment relative to tTA control from two independent experiments is reported. **G.** Luciferase activity of lysates from cells expressing tTA or Tat_SF2_ and transfected with an Egr1-luciferase reporter vector. Firely luciferase activity was normalized to Renilla luciferase activity and results are reported as fold induction relative to cells treated with ns siRNA in the presence of tTA.

We next investigated the contribution of PP2A to Tat-induced apoptosis using siRNA-mediated knockdown of the PP2A subunits. Jurkat cells were transfected with short interfering RNA (siRNA) targeting PPP2R1B, PPP2R5E or nonspecific siRNA and then infected with Ad-tTA or Ad-Tat_SF2_+Ad-tTA. mRNA levels of PPP2R1B and PPP2R5E were reduced by the PPP2R1B and PPP2R5E-specific siRNAs, respectively, but not by the control siRNA 24 hrs after infection (Figure 4A). The siRNAs targeting the PPP2R1B and PPP255E transcripts also reduced the corresponding protein levels (Figure 4C). Control siRNA had no effect. Levels of both PP2A subunits and of FOXO3a increased after Ad-Tat_SF2_ infection (Figure 3D and 4C). Reduction of PP2A subunits induced by the siRNAs resulted in reduced FOXO3a mRNA and protein (Figure 4A and 4C). Transfection of PP2A subunit siRNAs reduced expression of some of the FOXO3a target genes upregulated after Ad-Tat_SF2_ infection (Figure 4B). Furthermore, the reduction of PPP2R1B and PPP2R5E subunits also resulted in an increase of phosphorylated Akt1 and phosphorylated FOXO3a, supporting the role of the PP2A subunits in the accumulation of transcriptionally active, non phosphorylated FOXO3a and of its target genes (Figure 4C). We evaluated the role of the inhibition of PP2A on the expression of Egr-1, GADD45A and TRAIL, three FOXO3a target genes in Tat-expressing cells [1]. Treatment with siRNAs that target PPP2R1B and/or PPP2R5E reduced expression of Egr-1, GADD45A and TRAIL. The PPP2R1B siRNA had a more pronounced effect on the levels of FOXO3a and pFOXO3a proteins and of Egr-1 mRNA than did treatment of cells with PPP2R5E siRNA. (Figure 4A, 4B, and 4C).

As we previously linked the Tat-mediated increase of FOXO3a to apoptosis, we evaluated the impact of PP2A subunit siRNAs on Tat-induced apoptosis. siRNAs that target PPP2R1B and/or PPP2R5E in Jurkat cells after Ad-Tat_SF2_ infection caused a statistically significant decrease in apoptosis (Figure 4D). The same result was observed with okadaic acid, a PP2A inhibitor, but not with Akt1-1/2 or LY294002, an Akt and a PI3K inhibitor (Figure 4E). These experiments support a direct role of the PP2A subunits, but not of the above kinases, in Tat-mediated apoptosis.

The effect of siRNA-mediated inhibition of PPP2R1B in cells that do or do not express Tat was also evaluated by FOXO3a ChIP and amplification of promoters usually targeted by FOXO3a. Cells were transfected with PPP2R1B siRNA or control siRNA and then infected with Ad-Tat_SF2_. Egr-1, TRAIL, and GADD45A promoters were amplified by PCR using the DNA recovered after FOXO3a ChIP. As expected, the amounts of promoter sequence DNA detected by PCR were increased when the anti-FOXO3a immunoprecipitations of chromatin lysates were performed using Tat-expressing cells compared to cells infected with tTA alone and transfected with ns siRNA (Figure 4F) In contrast, PPP2R1B siRNA treatment was associated with reduced recovery of the same promoter DNAs. Taken together, these data indicate that Tat-induced PP2A subunits can affect transcriptional upregulation of FOXO3a-dependent genes by increasing the amount of transcriptionally active FOXO3a and its binding to the promoters of target genes.

To further evaluate whether the detection of FOXO3a at the Egr-1 promoter plays a role in the transcriptional regulation of Egr-1, we used a dual luciferase reporter assay. HeLa cells were transfected with an Egr-1-luciferase vector with or without FOXO3a siRNA for 24 hours, followed by infection with Ad-Tat_SF2_. Tat_SF2_ expression increased activity of the Egr-1 promoter by approximately four-fold compared to the control (Figure 4G). In contrast, transfection with FOXO3a siRNA reduced Tat-induced Egr-1 promoter activity in HeLa cells (Figure 4G). Similar results were observed when the experiment was carried out with 293T cells transfected with FOXO3a siRNA. Treatment with FOXO3a siRNA reduced expression of both Egr-1 and PTEN mRNA in HIV-infected HeLa cells [1]. Thus Tat can indirectly stimulate Egr-1 promoter activity by increasing the levels of transcriptionally active FOXO3a, which in turn can associate with the Egr-1 promoter. Increased trascription of Egr-1 further stimulated PTEN gene expression.

### Tat association with the PTEN promoter

**Figure 5:**
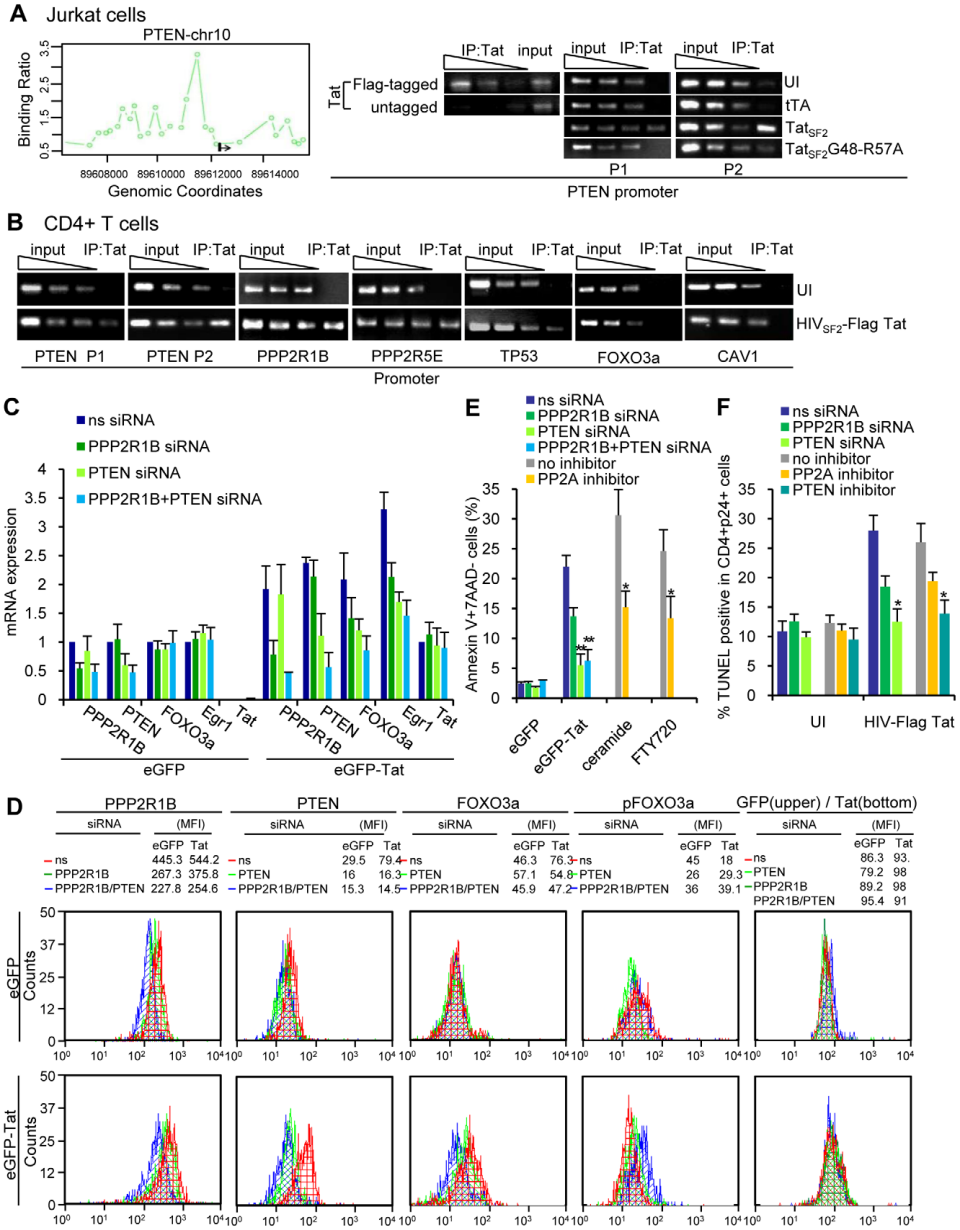
Tat association with the PTEN promoter. **A.** PTEN promoter enrichment ratio (ChIP DNA versus total genomic DNA in ChIP-Chip analysis) in Jurkat cells expressing Tat_SF2_ (first panel). ChIP analysis of the PTEN promoter in Jurkat T cells: two fold dilutions, starting from 5 ng, of DNA immunoprecipitated with anti-Flag antibody from samples expressing Tat_SF2_ with or without FLAG or 10 ng of input DNA, used as positive control, were amplified by PCR (first gel). In the second and third gel the signals obtained by PCR carried out with input DNA (90, 30, 10 ng of DNA) or with 3 ng of DNA extracted from immunoprecipitated samples are shown. Tat_SF2_G48-R57A was used as an additional negative control. P1 and P2 indicate 2 different sets of primers (Supplemental Table S3). **B.** ChIP analysis of the different cellular promoters in primary CD4+ T cells infected with a replication competent HIV expressing a flagged Tat. The signals obtained by standard PCR carried out with input DNA (90, 30, 10 ng of DNA) or with 1–5 ng of DNA extracted from immunoprecipitated samples are shown. The results obtained with two sets of primers, P1 and P2, are shown for the PTEN promoter. **C.** siRNA-mediated knockdown of PPP2R1B and PTEN reduces PPP2R1B, FOXO3a and FOXO3a-dependent gene expression in CD4+primary T cells infected with eGFP or eGFP-Tat retroviruses. RT-PCR results are normalized to GAPDH and reported as fold induction relative to cells treated with ns siRNA. Two independent experiments are reported. **D.** Protein expression analysis of PPP2R1B, PTEN, FOXO3a, or pFOXO3a by flow cytometry in CD4+ T cells treated with PPP2R1B and/or PTEN siRNAs and infected with a retrovirus expressing GFP only or Tat and eGFP. Results are reported as MFI. **E.** Level of apoptosis 48 h after Tat expression in CD4+ T-cells transduced with ns siRNA or PPP2R1B and/or PTEN siRNAs, or in CD4+ T-cells exposed to no inhibitor or 100 nM okadaic acid (a PP2A inhibitor) and then treated with PP2A enhancers (ceramide or FTY720). The mean ±SEM of two independent experiments is reported. (*) indicates p<0.05, (**) indicates p = 0.001. **F.** Level of apoptosis 48 h after HIV infection in CD4+ T-cells transduced with ns siRNA, PPP2R1B and/or PTEN siRNAs, untreated, treated with 100 nM okadaic acid (a PP2A inhibitor) or 10 nM bpV(HOpic) (a PTEN inhibitor). The mean ±SEM of two independent experiments is reported, (*) indicates p<0.05.

PTEN mRNA is upregulated in HIV-1 infected primary CD4+ T lymphocytes and in Tat-expressing Jurkat T cells [1]. PTEN is a key regulator of the PI3K/Akt1 pathway and controls the phosphorylation status of Akt1 and, indirectly, FOXO3a [45], [46]. High levels of PTEN reduce Akt1 phosphorylation with consequent reduced phosphorylation of FOXO3a. Non-phosphorylated FOXO3a translocates into the nucleus where it is transcriptionally active and increases the expression of FOXO3a transcription-dependent genes involved in either the extrinsic or the intrinsic apoptosis pathway. Furthermore, FOXO3a can also stimulate its own transcription [47]. Tat_SF2_ association with the PTEN promoter was detected by the ChIP-Chip analysis (Figure 3A, 5A left panel) and by conventional ChIP analysis (Figure 5A, right panels) in Jurkat cells. Cells were infected with an Ad-Tat_SF2_ with or without a FLAG tag fused to the *tat* gene and PCR was used to detect the PTEN promoter in chromatin immunoprecipitated with an anti-FLAG antibody. An enriched PTEN promoter DNA fragment was detected in cells expressing FLAG-Tat_SF2_ but not in cells expressing Tat_SF2_ without FLAG (Figure 5A, right panels). No PTEN amplification was found either in DNA from cells expressing tTA alone or from cells expressing the mutant Tat_SF2_G48-R57A that lacks the NLS. Therefore Tat expression is associated with the PTEN promoter and with an increase in its transcription activity.

The PTEN coding sequence is mutated and the corresponding protein is inactive in Jurkat cells due to the frame-shift mutations in both PTEN alleles, which result in the truncation of PTEN within the C-terminal C2 domain and the rapid degradation of the truncated protein [29]. Therefore we would not expect that the lack of phosphorylation of FOXO3a in Jurkat is dependent on PTEN but only on PP2A, even though an increase of PTEN RNA is observed in these cells. However, association of Tat with the PTEN promoter may be relevant in other cell types where this protein is functional. PTEN mRNA expression is increased in primary CD4+ T cells infected with HIV [1] and in these cells PTEN is functional. Indeed, when the association was investigated by conventional ChIP in primary CD4+ T cells infected with a HIV virus in which the *tat* gene was tagged with a FLAG epitope (Figure 5B), or a Tat expressing retrovirus (not shown), Tat could be found associated with the PTEN, PP2R1B, PPP2R5E, and TP53 promoters as detected by ChIP-Chip analysis in Jukat cells (Figure 5B). As in Jurkat cells, Tat did not associate with the FOXO3a promoter, excluding a prominent role for Tat in the direct transcriptional activation of FOXO3a observed in HIV-infected and Tat-expressing cells [1], or CAV1 promoter, used as a negative control.

To evaluate the role of PTEN in FOXO3a phosphorylation in primary CD4+ T cells, we investigated the effects of inhibition of PP2A and PTEN on FOXO3a and its target genes. We transfected primary CD4+ T cells with siRNA for PPP2R1B and/or PTEN for 24 hours and then infected them for 48 hours with a retrovirus expressing Tat and eGFP or eGFP alone as a control (Figure 5C). This treatment reduced expression of PPP2R1B and PTEN to approximately 60% of its original level. Reduction of PPP2R1B and PTEN expression resulted in reduced FOXO3a and Egr-1 mRNAs and protein expression and in an increase of p-FOXO3a (Figure 5C and 5D). Treatment with PTEN siRNA or the combination of PTEN and PPP2R1B siRNAs showed a significant reduction of apoptosis (p<0.001) in Tat expressing primary CD4+ T cells (Figure 5E). Treatment with PPP2R1B siRNA alone reduced apoptosis to approximately 46% (non statistically significant). This partial result was not surprising, considering that the siRNA treatment did not result in a complete knockdown of PPP2R1B and that PTEN remained active under these conditions. No synergy was apparent when PTEN and PPP2R1B siRNAs were introduced together, compared to transfection of PTEN siRNA alone. Tat-mediated PTEN activation may play a more significant role than PP2A in FOXO3a transcriptional activation in primary CD4+ T cells compared to Jurkat cells. Primary CD4+ T cells and Jurkat cells show concordant responses. PTEN, in addition to PP2A, is critical for Tat-mediated apoptosis of primary CD4+ T cells. When cells are exposed to a PP2A activator (ceramide or FTY720) apoptosis is induced (Figure 5E, grey bar). Subsequent treatment of these cells with okadaic acid (a PP2A inhibitor) showed a significant reduction of apoptosis (p<0.05) (Figure 5E, yellow bar). Level of apoptosis 48 hr after HIV infection in CD4+ T-cells transduced with PPP2R1B and/or PTEN siRNAs, or treated with a PP2A inhibitor or a PTEN inhibitor were significantly reduced compared to HIV infected cells treated with ns siRNA or not treated with any compound (Figure 5F, p<0.05).

### Effect of exogenous Tat on the regulation of the PI3K-PTEN-Akt pathway in primary CD4+ T cells

**Figure 6:**
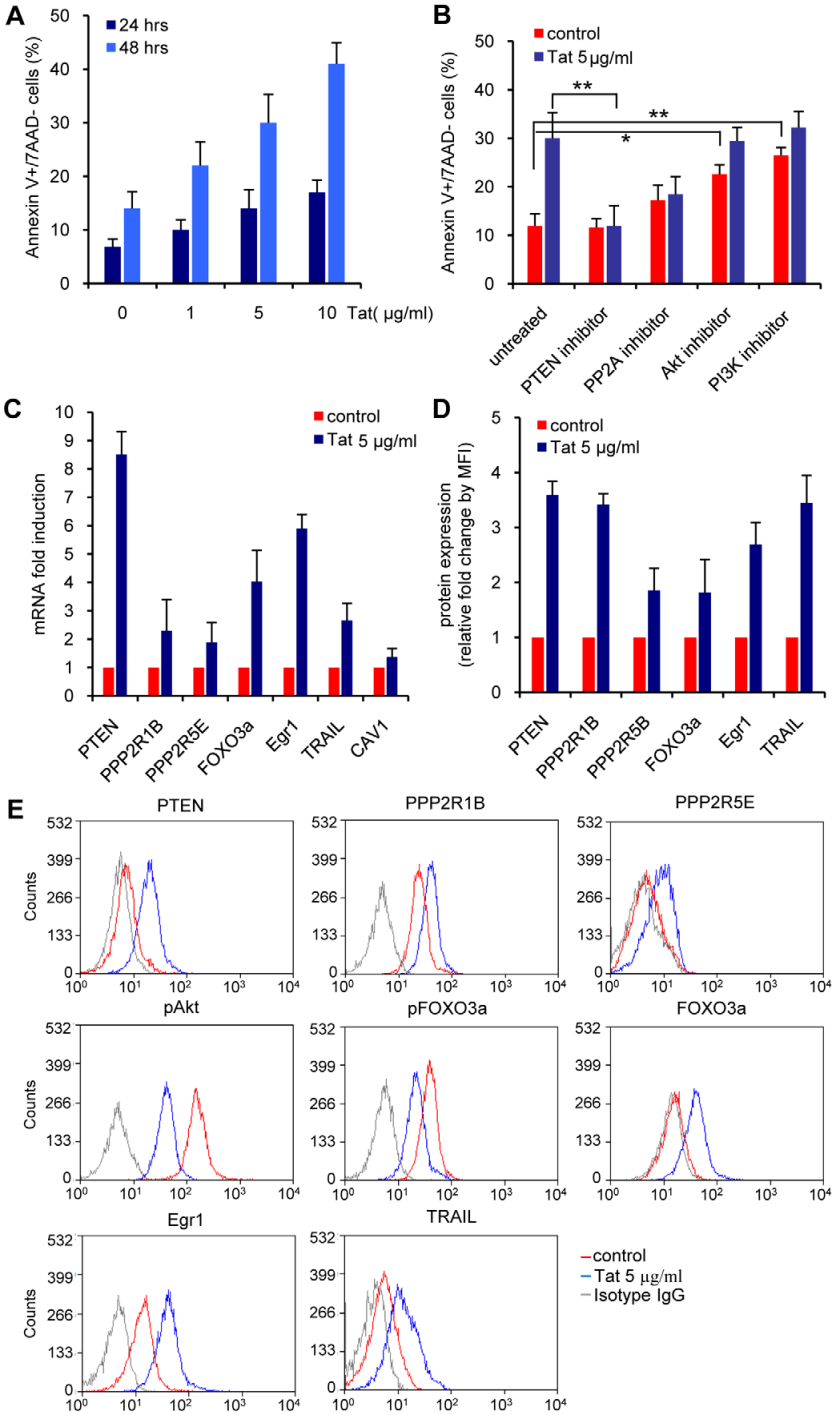
Apoptosis and PI3K pathway modulation in primary CD4+ T cells exposed to exogenous Tat. **A.** Levels of apoptosis 24 and 48 hours after exposure to different Tat concentrations in the medium. **B.** Levels of apoptosis 24 and 48 hours after exposure to 5 µg/ml of Tat and inhibitors of PTEN (10 nM bpV(HOpic)), PP2A (100 nM okadaic acid), Akt (50 µM Akt1-1/2), and PI3K (10 nM LY294002). The means ± SEM of three experiments are shown. (*) indicates p<0.05, (**) indicates p = 0.001. **C.** RNA expression levels of different genes part of the PI3K pathway in primary CD4+ T cells exposed to exogenous Tat. **D.** Corresponding protein levels for the same genes that are part of the PI3K pathway in primary CD4+ T cells 24 hours after exposure to exogenous Tat. RT-PCR results are normalized to GAPDH and reported as fold induction relative to cells untreated control. The average and SEM of three independent experiments is reported. **E.** One representative flow cytometric protein analysis related to the data reported in D. Results are reported as fold increase of mean fluorescence intensity (MFI) relative to the untreated control.

Exogenous Tat can be taken up by cells in cultures and transactivates HIV-1 LTR, if present in the cells, or induce apoptosis when the cultured cells are CD4+ T [17], [48]. Because of these observations, the possibility has been raised that HIV infected cells could release Tat, which has been detected in the serum of infected individuals [15], [16], and affect uninfected cells. To test the effect of exogenous Tat on the apoptosis of primary CD4+ T cells, recombinant Tat was added to the cultures of PBMC (Figure 6). Levels of early apoptosis were measured by evaluating the number of Annexin V+/7AAD- cells. An increase in apoptosis was observed in CD4+ T cells exposed to recombinant Tat in a dose- and time- dependent manner compared to the untreated control culture (Figure 6A). We also evaluated whether the exposure to recombinant Tat induced apoptosis by altering the PI3K-PTEN-Akt pathway (Figure 6B). PBMC were pretreated with inhibitors of PTEN (10 nM bpV(HOpic)), PP2A (100 nM okadaic acid), Akt (50 µM Akt1-1/2), and PI3K (10 nM LY294002) for 30 min before exposure to 5 µg/ml of recombinant Tat for 24 hr. Treatment with either PTEN or PP2A inhibitors showed a significant reduction of apoptosis in CD4+ T cells exposed to recombinant Tat (Figure 6B). In contrast, treatment with either the Akt or PI3K inhibitor in the absence of Tat increased apoptosis of CD4+ T cells, confirming the critical role of PI3K-Akt pathway in regulating cell growth and survival. Inhibition of Akt or PI3K in Tat expressing cells did not further increase the amount of apoptosis observed with Tat expression alone of CD4+ T cells, excluding the possibility of the involvement of other pathways in Tat-mediated apoptosis. These experiments support the concept that uninfected CD4+ T cells can undergo apoptosis when exposed to Tat released by HIV infected cells and that Tat induces apoptosis in a similar manner, whether expressed endogenously or present exogenously.

We next validated if exposure to exogenous recombinant Tat modulates the same cellular genes that were found up-regulated in CD4+ T cells expressing Tat endogenously (Figure 6C and 6D). PTEN, PPP2R1B, PPP2R5E, FOXO3a, Egr-1, and TRAIL RNAs were induced by exposure to recombinant Tat (Figure 6C). We also found a corresponding increased accumulation of PTEN, PPP2R1B, PPP2R5E, FOXO3a, Egr-1, and TRAIL proteins in the same cells (Figure 6D and 6E). These data confirm that exposure to exogenous Tat modulates the PI3K-PTEN-Akt pathway in primary CD4+ T cells in the same fashion as in CD4+ T cells expressing Tat endogenously.

### PTEN, PPP2R1B and PPP2R5E promoter sequences confer Tat-stimulated transcription on reporter gene

**Figure 7:**
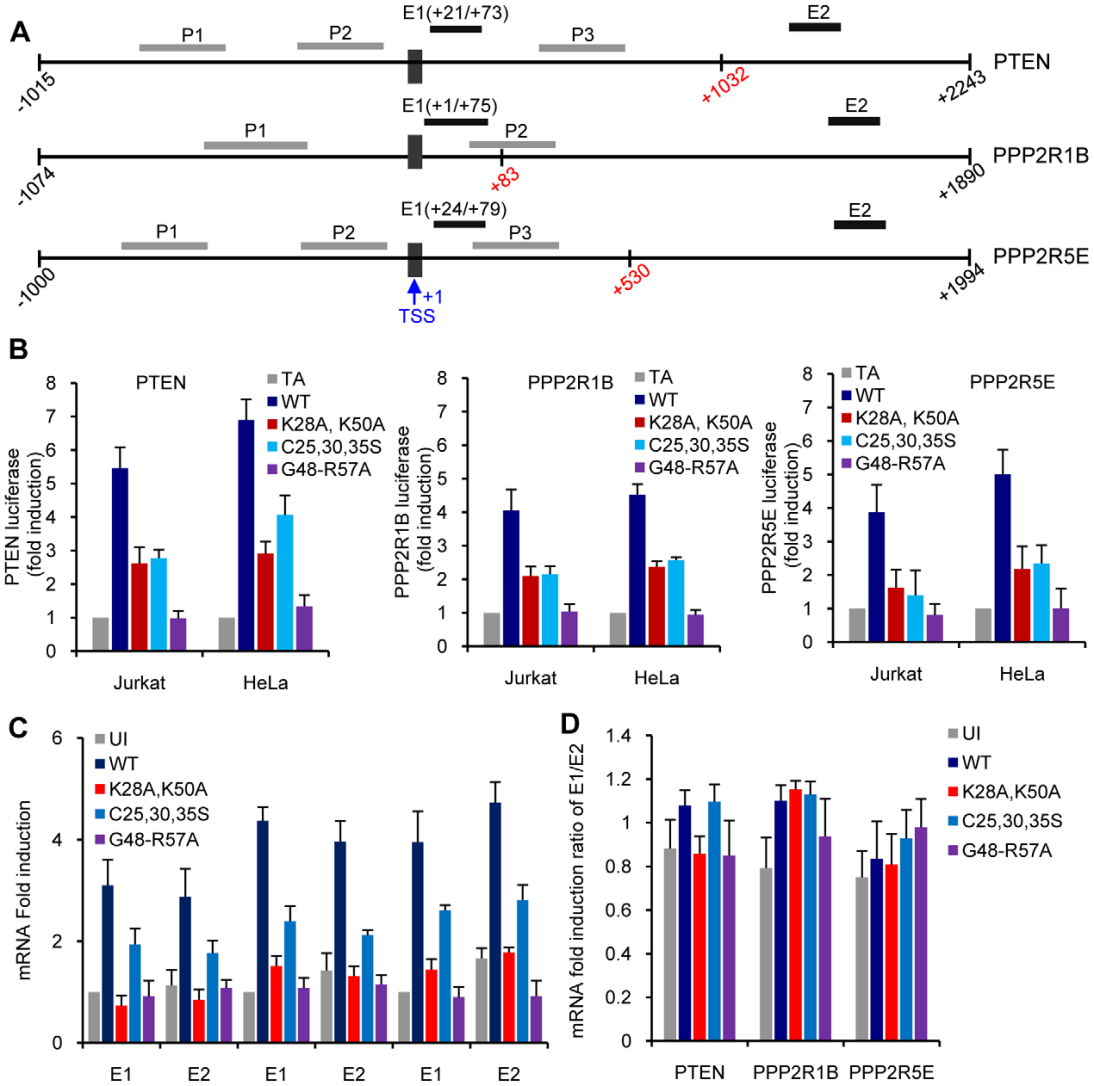
Analysis of transcription initiation and elongation at the PTEN, PP2R1B and PPP2R5E promoters. **A.** Schematic illustration of the location of amplified DNA (P1, P2, and P3) and RNA (E1, E2) fragments in the linear sequence of PTEN, PP2R1B and PPP2R5E genes. The transcriptional start site (TSS) is marked with a black box. Numbers in red mark the first nucleotide of the initiation codon. The promoter fragments used in B. start at the beginning of the individual lines and go to nucleotide −1. **B.** Luciferase activity in cells expressing Tat or Tat mutants and a luciferase gene under the control of PTEN (−1015 to−1), PPP2R1B (−1074 to−1), or PPP2R5E (−1000 to −1) promoters. **C.** Detection of PTEN, PP2R1B and PPP2R5E mRNA transcripts using primers in close proximity of (E1) or distant from (E2) the transcription start site. **D**. mRNA fold induction ratios between E1 and E2.

To investigate whether the PTEN, PPP2R1B and PPP2R5E promoter sequences bound by Tat would confer Tat-dependent stimulation of transcription on a reporter gene, ∼1000 bases of the PTEN (-1015 to-1), PPP2R1B (-1074 to-1), or PPP2R5E (-1000 to -1) promoters located 5′ of the transcriptional start site were introduced upstream of the luciferase gene. With all three promoters, the presence of wild type Tat increased luciferase activity relative to that obtained in the absence of Tat or Tat_SF2_G48-R57A (Figure 7B). Luciferase activity was intermediate when measured in cells expressing Tat_SF2_C25,30,35S or Tat_SF2_K28A,K50A (Figure 7B). These results confirm that the transcriptional activity of these promoters can be stimulated by Tat, and suggest that both Tat cofactor-binding domains are necessary for full activity.

When initiating transcription at the HIV promoter RNA polymerase II generates short RNA species and then pauses at the TAR element of the RNA transcript. Tat recruitment of P-TEFb at TAR releases paused polymerase and allows for accumulation of full length HIV mRNAs. [8], [9], [10], [11], [12]. We searched for evidence that RNAP polymerase II may pause in newly initiated PTEN, PPP2R1B and PPP2R5E transcripts by using RT-PCR to examine the steady-state levels of RNA species within the first 100 bases from the transcriptional start site (E1) and within the coding region of these genes (E2) (Figure 7C). The results did not reveal significant differences in the ratio of accumulation of short compared to long mRNAs in the presence or absence of Tat (Figure 7C, right panel). These results suggest that the increase in PTEN, PPP2R1B and PPP2R5E levels stimulated by Tat is unlikely to be caused by the release of RNA PolII that pauses after generating a short transcript, as it occurs during HIV transcription. Nonetheless, it is possible that Tat does facilitate pause release at the initiation start site or at a site within the first 80 nucleotides of the RNA transcript of PTEN, PPP2R1B and PPP2R5E genes. The lower amounts of Luciferance activity detected when transcription is dependent from the PTEN, PPP2R1B, or PPP2R5E promoters and of accumulated PTEN, PPP2R1B, or PPP2R5E RNA transcripts detected in the presence of Tat_SF2_C25,30,35S or Tat_SF2_K28A,K50A compared to wild type Tat support a role for Tat in recruiting factors that affect the processivity of PolII at the transcription start site of these promoters.

**Figure 8:**
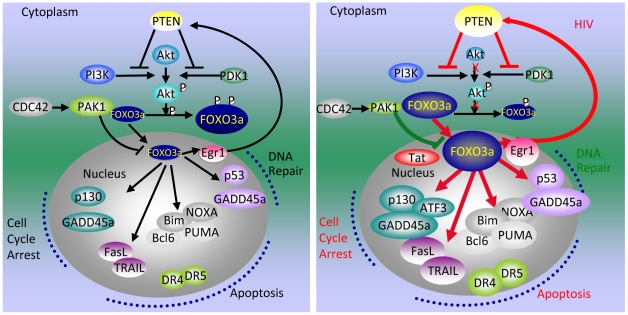
Tat-mediated alteration of apoptotic pathways regulated by FOXO3a. Red lines indicate the association of the protein in the circle at the beginning of the line with the promoter of the factor at the end of the line and its increased transcription. Red arrows connect proteins whose level is increased by the factor indicated at the beginning of the line. The green line that connects PAK1 to FOXO3a indicates reduced levels of PAK1. A red X marks a step that is significantly reduced.

In summary, Tat-mediated activation of apoptotic pathways in T cells starts with the association of Tat with promoters of phosphatase-encoding genes such as PTEN and PP2A. Increased transcription of phosphatase genes leads to reduced phosphorylation of Akt1 and of FOXO3a, its nuclear translocation, followed by the transcriptional activation of its own promoter and of FOXO3a target genes that affect of both the intrinsic and extrinsic apoptotic pathways. FOXO3a increases the transcription of Egr-1, one of its target genes, which further stimulates the transcription of PTEN, thereby reinforcing the pathway that leads to FOXO3a transcriptional activation (Figure 8).

## Discussion

Association of Tat with the PTEN and PP2A regulatory subunit promoters in T cells is the Tat-mediated event that leads to the activation of FOXO3a and its target genes, many of which are proapoptotic. Inhibition of these phosphatases leads to reduce apoptosis in T cells expressing Tat. A role for PP2A and PP1 in Tat-mediated regulation of HIV transcription was previously reported, but these reports did not investigate the role of Tat-phosphatase interactions in activation of cellular gene expression [49], [50], [51]. A previous investigation proposed that apoptosis in HIV infected CD4+ T cells results from the microtubule perturbation induced by the increased expression of Bim [52]. This mechanism is consistent with our observations, as Bim upregulation was detected during HIV infection in CD4+ T cells and in Tat expressing Jurkat cells [1]. This modulation resulted from the Tat mediated upregulation of trascriptionally active FOXO3a, which regulates Bim gene expression.

We failed to detect association of Tat with the FOXO3a or the Egr-1 promoters. However the level of expression for these genes falls when siRNA for PTEN and/or PP2A are introduced into Tat-expressing cells and partially inhibit expression of these genes. Transcriptionally active FOXO3a, which is also known to stimulate its own transcription [47], is thus linked to the activity of the phosphatases rather than to a closer interaction of Tat with the FOXO3a promoter. However, inhibition of SIRT-1 by Tat [14] might also increase the activity of FOXO3a, as SIRT-1 deacetylase activity can repress the activity of FOXO3a [53]. The detection of FOXO3a at the Egr-1 promoter indicates that Egr-1 is also a transcriptional target of FOXO3a and its increased transcription levels are likely to be directly dependent on the increase of transcriptionally active FOXO3a. The increased levels of Egr-1, which regulates transcription of PTEN [4], [5], [6] and is present at the PTEN promoter as shown by ChIP/PCR analysis, may further elevate the levels of PTEN, and so reinforce the circuit that leads to FOXO3a-mediated transcription.

Inhibition of expression of PTEN and PP2A in primary CD4+ T cells and of PP2A in Jurkat cells in the course of Tat expression reduced transcriptionally active FOXO3a and afforded protection from Tat-induced apoptosis (Figure 5 and 6). This confirms their role in apoptosis, as seen also for FOXO3a and Egr-1 inhibition [1]. Because we failed to detect a direct association of Tat with the promoters of these genes, these experiments point at PTEN or, in its absence, to PP2A, as a Tat target and initiatior of the apoptotic cascade. Inhibition of PPP2R1B did not reach statistical significance in reducing apoptosis in primary CD4+ T-cells, while statistical significance was attained when a siRNA against PTEN was used. It is possible that the activity of PTEN by itself is sufficient to initiate the apoptotic cascade and therefore inhibition of the PP2a subunit alone is not sufficient to drastically reduce apoptosis. Perhaps PTEN expression is more efficiently attenuated by siRNA compared to PPP2R1B, or PTEN governs the regulation of FOXO3a in these cells.

In regulating transcription from the HIV LTR, Tat's major role is in relieving RNAPII pausing at the TAR element by recruiting P-TEFb [8]. Because PTEN and PP2A transcription occurs in the absence of Tat in normal cells, we did not expect that Tat would significantly increase the transcription of these genes by relieving RNAPII pausing at the RNA level. Our data argue that the levels of transcripts that include the first 80 nucleotides occur at the same level as transcripts that are extended through the body of the gene (Figure 7C), but do not exclude the likely possibility that much shorter and unstable transcripts are produced by RNA polymerase II pausing. Because RNA polymerase II pausing now appears to occur very near the start site at all genes [54], it seems likely that Tat binding to these genes increases pause release and RNA polymerase II processivity by recruiting P-TEFb. Furthermore, other transcription factors that bind P-TEFb, such a c-Myc, increase transcript levels by using this mechanism [54]. In addition, the ability of Tat to increase transcription from PTEN and PP2A subunit promoters may depend on Tat facilitating P-TEFb transition form the inactive to the active form [55], [56]. Transcription of luciferance form PTEN and PP2A subunit promoter sequences located upstream of the transcription start site was not as significantly enhanced when a Tat mutant that does not bind P-TEFb was cotransfected with the reporter construct. The same was true when a Tat mutant that does not bind p300/was used in a similar experiment (Figure 7). It has been previously shown that Tat competes with HEXIM1, an inhibitor of P-TEFb activity [57], to increase the active pool of P-TEFb [58]. Tat therefore may act as a factor that increases RNAPII processivity by facilitating the transition of P-TEFb to its transcriptionally active form. Investigation of the details of this step will be the focus of future investigation.

Tat was found associated with 450 promoters during its expression in T cells. We do not know what specifically directs Tat to a subset of all promoters or if Tat associates with all of these promoters also in the context of HIV infection. Our current models are 1) the 450 promoters share one or more transcription factors that bind and recruit Tat to these promoters, and 2) Tat-bound P-TEFb is preferentially recruited to the 450 promoters by various transcription factors. These models will be investigated in future studies.

Levels of phosphorylated FOXO3a correlate with the survival of memory CD4+ T cells pFOXO3a is reduced in HIV-infected individuals and is higher in untreated, infected subjects with undetectable viremia [19]. These data support the *in vivo* relevance of our data, and suggest that regulating FOXO3a transcriptional activity may provide a target to control CD4+ T-cell apoptosis in vivo. Inhibition of PTEN and PP2A could in principle be beneficial to prevent HIV-mediated T cell apoptosis. However the role of inactive PTEN in many types of cancer is a serious impediment to this approach [6], [45]. Instead, inhibition of Tat may be a viable option to reduce HIV-induced apoptosis, especially in non-infected cells. Efforts aimed at Tat inhibitor discovery have met with limited success, because the primary endpoint was inhibition to reduce viremia. This is probably a poor endpoint because the role of Tat activity in HIV transcription is somewhat redundant and can be replaced by other factors. However, Tat remains a valuable drug target to reduce its deleterious effects on T cell survival. Antibodies against Tat, developed in vaccinated or infected patients, or drugs blocking Tat activity could neutralize Tat released from infected cells and prevent the protein to enter non-infected cells or to block its activity inside uninfected cells and prevent apoptosis of this population. While it is not known how much of the CD4+ T cell death in uninfected cells is due to this mechanism, it would be important to investigate in an experimental setting if these strategies offer some benefit.

## Materials and Methods

### Plasmids and transfection

Vectors for production of retroviruses (pCMMP-eGFP, pMMP-Tat1b-IGFP, pHDM-G/VSV-G envelope, and pMD.MLVgp) were generously provided by Dr. Jeng-Shin Lee from the Harvard Gene Therapy Initiative. Recombinant adenoviruses: Ad-tTA, Ad-Tat_SF2_, Ad-FLAG-Tat_SF2_, Ad-FLAG-Tat_SF2_G48-R57A, Ad-FLAG-Tat_SF2_C25,30,35S, and Ad-FLAG-Tat_SF2_K28A,K50A were constructed according to previously published procedures [59]. The Tat coding region was cloned into the vector pAd-TRE-MCS1. Tat is under a tetracycline-inducible promoter and co-infection with Ad-tTA, which expresses the tetracycline responsive transactivator, is required. DNA transfection in 293T cells was carried out by calcium phosphate precipitation. Supernatants containing viruses were prepared as described before [1].

### Cell lines, primary cells, treatments, Abs, and reagents

Human PBMCs were obtained from healthy donors from the Children's Hospital Boston blood bank. The purity of CD4+ lymphocytes isolated using a CD4+ T cell negative selection kit (Miltenyi Biotec) was more than 95%. All antibodies and siRNAs were previously described [1], except the human anti-PPP2R1B, anti-PPP2R5E antibodies (Santa Cruz Biotechnology), the PPP2R1B, PPP2R5E, siRNAs (Santa Cruz Biotechnology), and the PTEN siGENOME SMARTpool siRNA (Dharmacon).

### Transduction of T lymphocytes, infection of cell lines and siRNA treatments

T cells were exposed to the virus-containing supernatants of different vesicular stomatitis virus-G protein (VSV-G)-pseudotyped vectors [enhanced GFP (eGFP) or Tat-GFP] at multiplicity of infection (MOI) of 5 supplemented with 4 µg/ml protamine sulfate for 2 h. Jurkat T cells were exposed to adenoviruses at MOI of 20. Forty-eight hours after transduction T cells were sorted and GFP-positive cells were collected for PCR analysis of the RNA. All siRNAs were transfected into exponentially growing cells or electroporated at a final concentration of 3 µg into stimulated CD4+ T lymphocytes as previously described [1]. For luciferase reporter assay, HeLa and 293T cells were transiently transfected with nonspecific control or FOXO3a siRNA for 24 h and then infected with adenoviruses expressing tTA or Tat_SF2_ for 24 h. Cell lysates were used for luciferase reporter assay according to manufacturer's instructions.

### RNA isolation and quantitative real-time RT-PCR

The isolated RNA (100 ng) was reverse transcribed using an iScript cDNA kit (Bio-Rad) followed by amplification of HIV-1 Tat or cellular genes using SYBR Green SuperMix with ROX kit (Bio-Rad) and primers specific for the genes of interest and GAPDH as a control. Supplemental Table S2 reports the list of primers used in the amplification.

### Immunofluorescence staining and flow cytometry

Isolated CD4+ T lymphocytes (1×10^6^ cells) were prepared for intracellular staining as described before [1]. For intracellular staining, antibodies against PPP2R1B, pFOXO3a, and FOXO3a, were directly labeled with APC-Cy7, APC, or PE-Cy5 Tandem Conjugation Kit (Innova Biosciences). Flow cytometric acquisition was performed on MoFlow (Dako). Cell analysis was performed on the gated live-cell populations using Summit software (Dako). For apoptosis staining, CD4+ T lymphocytes or Jurkat cells were stained with Annexin V-APE and 7AAD and analyzed by flow cytometry as previously described.

### Confocal microscopy analysis

Ad-tTA or Ad-Tat_SF2_ infected cells were fixed and incubated first with a mouse monoclonal anti-PPP2R1B (Santa Cruz Biotechnology), or a rabbit polyclonal anti-FOXO3a, or anti-phospho-FOXO3a antibodies (Cell Signaling Biotechnology), and then with a FITC-conjugated goat anti-mouse IgG or TR-red- conjugated goat anti-rabbit IgG (Santa Cruz Biotechnology). Cells were stained with DRAG5 to visualize the nucleus (blue). The coverslips were mounted using ProLong Gold antifade reagent with DAPI (Molecular probes).

### Chromatin immunoprecipitation (ChIP) and DNA microarray analysis (ChIP-Chip)

ChIP was performed as previously described [60]. Briefly, 5×10^7^ cells were infected with adenoviruses, subsequently cross-linked, and sonicated to yield an average DNA fragment of 500 bps. The sheared chromatin was incubated with protein G magnetic beads (Invitrogen) coupled to anti-FLAG (Sigma M2), anti-p300 polyclonal, anti-FOXO3a polyclonal, and anti-Egr-1 polyclonal antibodies (Santa Cruz Biotechnology). These antibodies have been previously used in ChIP and do not have significant background [61], [62], [63]. Amplified DNA was labeled and purified using Bioprime random primer labeling kits (Invitrogen, immunoenriched DNA was labeled with Cy5 fluorophore, whole cell extract DNA was labeled with Cy3 fluorophore). Labeled DNA was mixed and hybridized to Agilent Human Promoter ChIP-on-chip Microarray Set for 20 hours at 40°C. The microarrays (Design ID - 014706 and 014707) have probes for about 17,000 human promoters. Arrays were then washed using standard Agilent protocols and scanned using an Agilent DNA microarray scanner BA. Scans were manually examined for abnormal features and intensities were extracted for each spot automatically using Agilent feature extraction software. We calculated the log of the ratio of median normalized intensity in the IP-enriched channel to median normalized intensity in the genomic DNA channel for each probe and used a whole chip error model [64] to calculate confidence values for each spot on each array (single probe p-value). This error model functions by converting the intensity information in both channels to an X score which is dependent on both the absolute value of intensities and background noise in each channel. The X scores for an array are assumed to be normally distributed which allows for calculation of a p-value for the enrichment ratio seen at each feature. To determine bound regions in the datasets, we calculated the average X score of the 60-mer and its two immediate neighbors. If a feature was flagged as abnormal during scanning, we assumed it gave a neutral contribution to the average X score. Similarly, if an adjacent feature was beyond a reasonable distance from the probe (based on the maximum size of labeled DNA fragments hybridized to the array), we assumed it gave a neutral contribution to the average X score. This set of averaged values gave us a new distribution that was subsequently used to calculate p-values of average X (probe set p-values). If the probe set p-value was less than 0.001, the three probes were marked as potentially bound. In addition, the three probes in a probe set must each have single probe p-values<0.05 or the center probe in the probe set has a single probe p-value<0.01 and one of the flanking probes has a single point p-value<0.1. Association with promoters was identified if the bound regions were 8 kb upstream or 2 kb downstream of the Transcription Start Site [64]. For conventional ChIP analysis the purified DNA was quantified by qPCR. The primers used in qPCR are described in Supplemental Table S3. For the qPCR reaction, 10 ng of immunoprecipitated material was used, and for whole cell extract DNA samples (or input) a range of DNA amounts (10–90 ng of DNA) were used. PCR products obtained by standard PCR for qualitative analysis were visualized on agarose gel with ethidium bromide.

### Protein phosphatase and protein phosphatase 2A (PP2A) activity

Protein phosphatase activity was measured using a commercially available Ser/Thr phosphatase assay kit (Upstate Biotechnology). Cell lysates were used to measure phosphatase activity using p-nitrophenyl phosphate (pNPP) or free phosphate released by the phosphatase from a specific peptide substrate KRpTIRR using the malachite green system. For protein phosphatase 2A (PP2A), total cellular protein lysate was incubated with protein A-agarose slurry with anti-PP2A-C (2 µg/ml, Upstate Biotechnology). A commercially available PP2A immunoprecipitation phosphatase assay kit (Upstate Biotechnology) was used to measure phosphate release as an index of phosphatase activity.

### Treatments with inhibitors

PBMC (1×10^6^ cells) were infected with HIV-Flag Tat (viral amount corresponding to 20 ng of p24) for 6 hours and then resuspended with fresh medium in the presence or absence of PP2A inhibitor (100 nM okadaic acid) or PTEN inhibitor (10 nM bpV(HOpic). A second dose of the inhibitors was added to the cultures after 48 h. The percentage of TUNEL+ or Annexin V+ cells was determined by fluorescence-activated cell sorter analysis on day 3 or 5. Jurkat T cells were exposed to adenoviruses at MOI of 20 in the presence or absence of inhibitors. After 2 h, cells were washed and fresh media was replaced. Level of apoptosis was expressed as the percentage of Annexin V+7AAD- 24 h after transduction. PBMC were incubated without or with PP2A inhibitor (100 nM okadaic acid) for 1 h and then treated with two PP2A enhancers, sphingolipid ceramide N-Deacyclase (Calbiochem, cat. #567704) (10 µM) or FTY720 (EMD, Gibbstown, NJ) (10 nM), for 48 h. Level of apoptosis was expressed as the percentage of Annexin V+CD4+. Recombinant Tat_HIV-Bal_ was added at the indicated concentrations into the cultures of PBMC in the presence or absence of PTEN, PP2A, Akt, and PI3K inhibitors (10 nM bpV(HOpic, 100 nM okadaic acid, 50 µM Akt1-1/2, 10 nM LY294002, respectively), which were added 30 minutes before the addition of Tat.

### Luciferase assays

HeLa and 293T cells were transfected with nonspecific siRNA or siRNA targeting FOXO3a with SureFECT transfection (SABiosciences). Luciferase activities were assessed using the dual-luciferase reporter assay system (Promega, Madison, WI). Firefly luciferase activity was normalized to the activity of the Renilla luciferase control.

### Immunoblots

Total proteins were separated by SDS-PAGE, transferred onto the nitrocellulose membrane, and incubated with Abs raised against FOXO3a, phospho-FOXO3a (Ser318), PTEN, phospho-Akt1 (Ser473), PPP2R1B, PPP2R5E, and PPP2C (Santa Cruz Biotechnology). The secondary Ab was detected with Pierce ECL substrate. The blots were exposed to Hyperfilm, and the signals were quantified by scanning densitometry with Molecular Analyst 1.5 software (Biorad). To account for any differences in loading, target band densitometries were divided by actin densitometry obtained in the same lane. These corrected densitometries were normalized to controls in each experiment.

### Statistical analysis

A two-tailed, two-sample Student t test was used to calculate p-values for differences in means between groups. The data are expressed as means ± SEM. For statistical inference, a p-value of <0.05 was considered significant.

## Supporting Information

Table S1 List of promoters enriched in Tat ChIP-Chip.
(0.13 MB PDF)

Table S2 Oligonucleotide sequences used for RT-PCR.
(0.06 MB PDF)

Table S3 Oligonucleotide sequences used for ChIP-qPCR and promoter fold enrichment.
(0.06 MB PDF)
