# Supplementary material for: Association of Tat with Promoters of PTEN and PP2A Subunits Is Key to Transcriptional Activation of Apoptotic Pathways in HIV-Infected CD4+ T Cells
Source: PLoS Pathog. 2010 Sep 16;6(9):e1001103. doi: 10.1371/journal.ppat.1001103 (PMC2940756; doi:10.1371/journal.ppat.1001103)
Supplement: Table S1 — List of promoters enriched in Tat ChIP-Chip. (0.13 MB PDF) [file ppat.1001103.s001.pdf]

Supplemental Table S1: List of promoters enriched in Tat ChIP-Chip

GeneSymbol	P-value	Binding Ratio
KCNMB4	0.00099	3.74
FLJ10357	0.00099	2.75
BUB3	0.00097	2.19
SLC29A3	0.00097	1.89
NECAP1	0.00093	2.17
ORMDL2	0.00092	2.58
CIP29	0.00092	2.58
HECTD2	0.00092	1.96
THAP7	0.00092	3.13
P2RXL1	0.00092	3.13
COL4A2	0.00092	2.73
WDR24	0.00091	2.04
LOC339123	0.00091	2.04
TBL3	0.00090	2.17
RPS2	0.00090	2.17
RNF151	0.00090	2.17
LOC286444	0.00090	2.17
FLJ25168	0.00090	3.51
MGC20255	0.00088	1.87
NBR1	0.00088	2.36
COL20A1	0.00088	2.60
DOK6	0.00087	3.38
NPM3	0.00086	2.23
FGFR1OP2	0.00085	3.25
C12orf11	0.00085	3.25
FLJ21749	0.00085	2.13
CREM	0.00084	15.79
NFS1	0.00083	1.97
C20orf52	0.00083	1.97
RAB34	0.00083	2.27
NEK8	0.00083	2.27
LOC116238	0.00083	2.27
VNN2	0.00080	4.70
SPAG7	0.00080	2.89
C20orf86	0.00080	2.69
PLDN	0.00077	1.89
ZNF23	0.00077	3.05
NP	0.00075	3.78
FEM1B	0.00075	2.39
NGRN	0.00072	2.63
RBM4	0.00072	2.32
RAB3C	0.00072	2.16
DGCR8	0.00072	1.79
HSPC023	0.00071	2.23
KCTD2	0.00071	2.01
ATP5H	0.00071	2.01
FLJ16331	0.00071	2.36
IFNGR2	0.00069	2.26
MPP3	0.00068	2.46
NEUROG3	0.00068	3.57
ZNF566	0.00068	2.45
RNF6	0.00066	3.12

EIF4B	0.00065	3.01
DDX50	0.00065	2.22
MRPL48	0.00062	2.55
ELAC2	0.00062	2.10
ARID2	0.00061	2.60
NDUFA6	0.00060	2.13
TST	0.00060	2.50
C14orf37	0.00058	3.07
NDRG4	0.00056	2.44
CLTC	0.00055	2.03
MGC51082	0.00055	2.17
RAB30	0.00055	1.95
SSTR1	0.00055	2.96
MGC20410	0.00054	2.74
RPL28	0.00052	2.22
ZNF232	0.00052	2.81
USP6	0.00052	2.81
MGC16824	0.00052	2.06
HNRPA1	0.00051	2.92
CBX5	0.00051	2.92
KIAA0247	0.00050	2.35
GPRC5A	0.00050	3.28
RNMTL1	0.00049	2.14
RAB8B	0.00048	2.38
AKAP8L	0.00048	1.90
SLC38A1	0.00048	2.51
ZCCHC10	0.00048	4.95
SLC13A2	0.00047	2.74
CCNL1	0.00046	2.15
ABC1	0.00045	2.59
PLXDC1	0.00045	2.61
CSE1L	0.00044	1.86
PTD012	0.00044	2.66
MGC5306	0.00044	2.66
CCNB3	0.00043	2.08
C17orf35	0.00043	2.21
WDR71	0.00043	2.34
E2IG2	0.00043	2.34
LOC399900	0.00042	1.86
PAF1	0.00042	2.24
IXL	0.00042	2.24
NPPA	0.00042	1.88
LOC90321	0.00042	2.33
HIRA	0.00041	3.01
ZNF213	0.00040	2.51
C14orf169	0.00040	4.10
TRIM34	0.00040	2.99
FLOT2	0.00039	1.74
TGIF	0.00039	2.28
ZNF480	0.00038	2.50
UBASH3A	0.00038	2.40
TMPRSS3	0.00038	2.40
SIAH1	0.00037	2.18
LMO1	0.00037	2.26
KIAA1623	0.00037	1.93

GTPBP3	0.00037	1.93
CDCA4	0.00036	2.63
LOC197322	0.00036	2.28
HIST1H1B	0.00036	3.51
WBP11	0.00036	2.69
MGC47869	0.00036	2.69
FLJ31153	0.00036	2.95
TENC1	0.00036	2.35
BLR1	0.00035	2.03
MAWBP	0.00035	4.00
HNRPH3	0.00035	4.00
C12orf2	0.00035	2.28
MGC2963	0.00035	2.70
ESCO1	0.00035	3.17
SPRED1	0.00035	2.49
COPS3	0.00034	2.58
PCNA	0.00034	2.53
CDS2	0.00034	2.53
C20orf30	0.00034	2.53
TUBA3	0.00034	2.36
SLC1A5	0.00033	3.70
FLJ10970	0.00033	2.92
SPAG9	0.00033	3.04
MAN2C1	0.00032	2.26
SI	0.00032	5.31
BDNF	0.00031	3.73
SMAP	0.00031	1.68
FLJ12476	0.00031	2.22
FLJ11506	0.00031	2.22
MLLT6	0.00030	2.32
TULP3	0.00029	2.71
TPM4	0.00029	2.28
DSCAML1	0.00029	4.42
DUT	0.00028	2.10
RPL27	0.00028	2.46
IFI35	0.00028	2.46
FLJ20643	0.00028	2.32
ALDH16A1	0.00028	2.32
C11orf30	0.00028	2.43
CAND1	0.00028	2.66
MAPK8IP3	0.00028	2.08
MON1B	0.00028	1.63
CASC5	0.00028	2.55
GDPD5	0.00027	2.46
TFPT	0.00026	1.49
PRPF31	0.00026	1.94
WDR68	0.00026	1.93
WIBG	0.00026	2.76
DGKA	0.00026	2.76
OIP5	0.00026	1.61
NUSAP1	0.00026	1.57
KIAA0652	0.00026	2.26
FLJ32675	0.00026	2.26
PLEKHF1	0.00025	4.26
MARCH3	0.00025	15.55

MYL6	0.00025	2.16
MLC1SA	0.00025	2.16
RND1	0.00025	2.91
ZDHHC6	0.00025	2.16
VTI1A	0.00025	2.16
VAMP1	0.00025	2.23
RPL3	0.00024	2.76
PFKM	0.00024	3.21
MRPS35	0.00024	2.25
C20orf31	0.00024	2.11
TARSL2	0.00024	2.94
ETNK1	0.00024	2.24
LOC283392	0.00024	2.67
EFHA1	0.00023	2.26
SPN	0.00023	2.72
EIF3S10	0.00022	2.74
CYB5R4	0.00022	6.67
SLC9A8	0.00022	2.28
RPL7	0.00022	4.79
RDH10	0.00022	4.79
LOC90193	0.00022	4.79
TUBB6	0.00021	1.83
GADD45B	0.00020	2.84
TRPM2	0.00020	1.77
PSMA6	0.00020	2.23
MGC13183	0.00020	1.97
POU4F1	0.00019	2.50
IFNAR2	0.00019	1.69
HNRPM	0.00019	2.92
ELAVL1	0.00019	2.92
ASAH2	0.00018	7.80
LSM4	0.00017	1.98
MN1	0.00017	3.73
C18orf23	0.00017	2.33
TMED3	0.00017	2.38
WDR74	0.00017	4.61
UBL4	0.00016	2.26
ZNF192	0.00016	1.68
CPNE1	0.00015	2.08
HIST1H1D	0.00015	4.26
ZNF160	0.00015	2.90
AQR	0.00015	3.11
RPS28	0.00015	1.60
NDUFA7	0.00015	1.60
PRKAG1	0.00015	2.50
GTF2H3	0.00014	2.23
FANCF	0.00014	2.62
C14orf112	0.00014	2.21
MC2R	0.00014	2.80
MEF2A	0.00014	2.52
FLJ12118	0.00014	2.56
MGC57359	0.00014	3.95
DKFZP586B1621	0.00014	2.13
DDB1	0.00014	2.13
PLCB1	0.00014	2.53

PANK1	0.00014	4.13
RPS9	0.00014	1.94
GOSR2	0.00013	2.44
ITPR2	0.00013	2.39
FLJ10979	0.00012	1.89
NDST2	0.00012	2.36
TEX14	0.00012	1.51
RAD51C	0.00012	1.52
PFAAP5	0.00012	2.42
LOC88523	0.00012	2.42
GANAB	0.00012	2.11
MUS81	0.00012	2.82
CFL1	0.00012	2.82
GRP	0.00012	2.64
CDH22	0.00011	4.11
GRINL1A	0.00011	3.04
GOSR1	0.00011	3.94
HSPA8	0.00011	2.43
OAZ1	0.00011	5.71
TTC3	0.00011	2.73
LOC286495	0.00011	2.73
DSCR5	0.00011	2.73
NR1H3	0.00011	2.96
ACP2	0.00011	2.96
MGC29649	0.00009	2.09
DKFZp762N1910	0.00009	2.09
BSCL2	0.00009	2.09
STK24	0.00009	3.73
ROM1	0.00009	3.53
MTA2	0.00009	3.53
C20orf3	0.00009	2.47
PDCD7	0.00009	3.08
ARHGDIB	0.00008	1.59
MRPL39	0.00008	2.66
ZFP36L1	0.00008	3.31
ING1	0.00008	2.83
GNB2L1	0.00008	4.37
CCT7	0.00008	5.75
C2orf7	0.00008	5.75
FLJ13213	0.00007	2.49
FLJ40342	0.00007	2.49
IRAK4	0.00007	2.24
DKFZP434G1415	0.00007	2.24
PSMC6	0.00007	3.03
CRSP6	0.00007	2.35
WNK4	0.00007	3.03
MGC10540	0.00007	3.03
DDIT4	0.00007	3.40
USP47	0.00007	3.39
SLC16A12	0.00007	2.19
CHURC1	0.00007	2.83
PANK2	0.00007	2.59
CCT6B	0.00007	3.15
CCDC16	0.00007	3.15
MGC29671	0.00007	2.60

DLAT	0.00006	2.00
ZNF382	0.00006	2.46
FLJ25590	0.00006	2.20
C21orf127	0.00006	1.94
SSRP1	0.00006	2.38
P2RX3	0.00006	2.38
MBD6	0.00005	2.89
DDIT3	0.00005	2.89
MGC11335	0.00005	2.61
PTEN	0.00005	3.46
TTBK2	0.00005	2.79
METTL3	0.00005	2.66
PRRG2	0.00005	2.29
NOSIP	0.00005	2.29
HTR2A	0.00005	2.59
APP	0.00005	2.65
RPS26	0.00005	3.24
LOC441377	0.00005	3.24
MGC39681	0.00005	2.81
DRD2	0.00005	3.52
RPS24	0.00005	2.42
POLR3A	0.00005	2.42
CX36	0.00004	3.14
LOC112869	0.00004	3.37
CNN2	0.00004	3.16
C19orf6	0.00004	3.16
ZNF502	0.00004	5.96
PFAS	0.00004	3.11
FLJ22170	0.00004	3.11
POLG	0.00004	3.00
DYRK4	0.00004	1.90
ASMTL	0.00004	2.60
OSBPL1A	0.00004	3.86
PPP2R5E	0.00004	2.52
TK2	0.00004	3.45
CKLFSF1	0.00004	3.45
CKLF	0.00004	3.45
TUBD1	0.00004	2.62
RPS6KB1	0.00004	2.62
TRIB3	0.00004	2.53
ZNF446	0.00003	2.05
STRA6	0.00003	2.49
PDE3B	0.00003	2.59
MGC10992	0.00003	2.29
TP53	0.00003	2.53
FLJ10385	0.00003	2.53
LRFN5	0.00003	2.17
RPS15A	0.00003	2.34
LOC440828	0.00003	2.34
EIF4A2	0.00003	4.84
HNRPUL1	0.00003	2.62
FLJ32800	0.00003	3.40
DTWD1	0.00003	3.40
ICAM5	0.00003	2.53
FLJ25059	0.00002	2.21

ELP4	0.00002	2.21
ARPP-19	0.00002	3.39
CHEK1	0.00002	1.97
DDX6	0.00002	2.43
UQCRC2	0.00002	1.87
SRRM2	0.00002	2.91
CDH8	0.00002	2.71
PTDSR	0.00002	2.34
LOC124512	0.00002	2.34
PFN1	0.00002	2.44
ENO3	0.00002	2.44
MTMR6	0.00002	2.75
CKLFSF5	0.00002	2.15
C21orf91	0.00002	2.70
ZNF207	0.00002	3.01
NJMU-R1	0.00002	3.01
C20orf6	0.00002	2.22
PXDNL	0.00002	2.96
RBM7	0.00002	2.92
FLJ20010	0.00002	2.92
ACTN4	0.00002	2.36
RPL21	0.00002	2.45
LOC440487	0.00002	2.45
LOC389156	0.00002	2.45
LOC388143	0.00002	2.45
RAD52	0.00002	2.75
SLC3A2	0.00002	3.36
RPL38	0.00001	2.43
HYPC	0.00001	1.66
ADCYAP1	0.00001	2.71
MLL	0.00001	1.65
MGC13053	0.00001	1.65
FLJ11783	0.00001	1.65
CA7	0.00001	3.97
RPS3	0.00001	2.00
LOC440991	0.00001	2.00
ANP32A	0.00001	2.93
ZNF271	0.00001	3.44
DNAJA2	0.00001	3.08
FLJ11151	0.00001	2.42
MAST1	0.00001	10.77
EIF4G2	0.00001	2.18
CUZD1	0.00001	3.31
MGC21518	0.00001	1.58
ZBTB4	0.00001	4.36
POLR2A	0.00001	4.36
TCF4	0.00001	2.34
MGC14151	0.00001	3.50
FLJ32499	0.00001	3.50
DCPS	0.00001	2.86
LDHB	0.00001	2.36
SNRPB	0.00001	3.80
NOL5A	0.00001	3.03
TXNL5	0.00001	3.19
KIAA0753	0.00001	3.19

CDC27	0.00001	2.26
KIAA1409	0.00001	2.73
BTBD7	0.00001	2.73
LOC440082	0.00001	3.61
SLC38A6	0.00001	3.18
KIAA1393	0.00001	3.18
CDC40	0.00001	15.57
C15orf23	0.00001	2.73
GTL3	0.00001	2.61
RPL3L	0.00001	2.50
MRPL51	0.00001	2.60
CNAP1	0.00001	2.60
USP52	0.00001	2.56
IL23A	0.00001	2.56
SLC17A6	0.00001	3.17
ZFYVE19	0.00001	1.64
DNAJC17	0.00001	1.64
HIST1H1C	0.00001	3.82
PPP2R1B	0.00001	5.13
WDR4	0.00001	2.85
TXNDC10	0.00001	2.33
DKFZP434K1421	0.00001	2.24
TBRG1	0.00001	1.61
SHMT2	0.00001	1.86
DHX34	0.00001	2.28
SYVN1	0.00001	2.19
ADAMTS1	0.00001	2.16
PSMD8	0.00000	3.37
TRIP4	0.00000	2.91
KIAA0101	0.00000	2.91
KIAA1404	0.00000	2.86
FUT4	0.00000	3.49
PRKCSH	0.00000	3.19
MGC20983	0.00000	3.19
DDX5	0.00000	3.57
GPKOW	0.00000	1.85
RPL23	0.00000	2.87
LOC442209	0.00000	2.87
TNKS1BP1	0.00000	2.89
C14orf149	0.00000	2.37
C14orf100	0.00000	2.37
PRG3	0.00000	2.67
RAB37	0.00000	4.20
CSTF2T	0.00000	2.18
VSX1	0.00000	4.10
SNAP25	0.00000	5.68
IFNAR1	0.00000	2.94
MSN	0.00000	3.01
EIF4A1	0.00000	3.04
BUCS1	0.00000	3.28
MNAT1	0.00000	2.91
MYCBP2	0.00000	2.72
TOB2	0.00000	4.19
RPL13A	0.00000	2.49
SORBS1	0.00000	3.16

ITPA	0.00000	4.98
C20orf116	0.00000	4.98
ETS1	0.00000	3.74
SAP18	0.00000	2.73
CECR5	0.00000	2.21
NUP160	0.00000	2.54
CDK3	0.00000	2.96
ACOX1	0.00000	2.96
ZC3H10	0.00000	3.61
RPL41	0.00000	3.61
STX10	0.00000	4.49
PDE4C	0.00000	3.67
BZRAP1	0.00000	6.15
