# Supplementary material for: Association of Tat with Promoters of PTEN and PP2A Subunits Is Key to Transcriptional Activation of Apoptotic Pathways in HIV-Infected CD4+ T Cells
Source: PLoS Pathog. 2010 Sep 16;6(9):e1001103. doi: 10.1371/journal.ppat.1001103 (PMC2940756; doi:10.1371/journal.ppat.1001103)
Supplement: Table S2 — Oligonucleotide sequences used for RT-PCR. (0.06 MB PDF) [file ppat.1001103.s002.pdf]

Supplemental Table S2: Oligonucleotide sequences used for RT-PCR

Gene	RefSeq no.	Primers	5' Oligo	3' Oligo	Size
PTEN	NM_000314	E1(+21/+73)<br>E2(+697/+859)	TCCCGTCCGCCTCTC<br>CGACGGGAAGACAAGTTCAT	GGGCGCCTCGGAAG<br>AGGTTTCCTCTGGTCCTGGT	53<br>163
PPP2R1B	NM_002716	E1(+1/+75)<br>E2(+1220/+1326)	GGCCTTCGTCCCTACTGC<br>TTGGAATCCGTCAGCTCTCT	CCTCCTGCTGCTGGTCA<br>CAGCAGCGGCATATACTCAA	75<br>107
PPP2R5E	NM_006246	E1(+24/+79)<br>E2(+1208/+1311)	AGCCGCCCGGTGATA<br>CTCGGAAGTCCGTCAGAAAA	GTGGGTCCCAGTCAATGC<br>TTTCTCACGCTGACGATCTG	56<br>104
Egr1	NM_001964	E1(+58/+115)<br>E2(+302/+501)	TTCGGATCCTTTCCTCACTC<br>CCGCAGAGTCTTTTCCTGAC	TCATCTCCTCCAGCTTAGGG<br>TGGGTTGGTCATGCTCACTA	58<br>200
TRAIL	NM_003810	E1(+38/+97)<br>E2(+278/+436)	GACAGACCTGCGTGCTGAT<br>TGAGAACCTCTGAGGAAACCAT	CAGCCACACAGAGAGACTGC<br>CCTTTTCATTCTTGGAGTTTGG	60<br>159
GADD45A	NM_001924	E1(+11/+60)<br>E2(+386/+491)	AGGAATTCTCGGCTGGAGAG<br>ATCCACATTCATCTCAATGGAAG	CCCCACCTTATCCATCCTTT<br>TCAGGGAGATTAATCACTGGAAC	50<br>106
FOXO3a	NM_201559	E1(+39/+104)<br>E2(+985/+1270)	GCTCGAAGTGGAGCTGGA<br>GCAAGCACAGAGTTGGATGA	AGGGGCCACGTACAGGAT<br>CAGGTCGTCCATGAGGTTTT	66<br>186
CAV1	NM_001753	E1(+44/+55)<br>E2(+179/+354)	TCTGGGGGCAAATACGTAGA<br>ACGATGACGTGGTCAAGAT	GGATGGGAACGGTGTAGAGA<br>GTAAATGCCCCAGATGAGTG	52<br>176
GAPDH	NM_002046	E1(+20/+72)<br>E2(+420/+532)	GAGTCAACGGATTTGGTCGT<br>TGACAACAGCCTCAAGATCA	GTTAAAAGCAGCCCTGGTGA<br>CTGTGGTCATGAGTCCTTCC	53<br>113
