# Supplementary material for: Association of Tat with Promoters of PTEN and PP2A Subunits Is Key to Transcriptional Activation of Apoptotic Pathways in HIV-Infected CD4+ T Cells
Source: PLoS Pathog. 2010 Sep 16;6(9):e1001103. doi: 10.1371/journal.ppat.1001103 (PMC2940756; doi:10.1371/journal.ppat.1001103)
Supplement: Table S3 — Oligonucleotide sequences used for ChIP-qPCR and promoter fold enrichment. (0.06 MB PDF) [file ppat.1001103.s003.pdf]

Supplemental Table S3: Oligonucleotide sequences used for ChIP-qPCR and promoter fold enrichment

Gene	Ref Seq no.	Primers	5' Oligo	3' Oligo	Average enrichment
PTEN	NM_000314	P1(-534/-353)<br>P2(-288/-176)<br>P3(+121/+239)	GGGGAATCTCTAGGCAAAGG<br>GCACCCATCTCAGCTTTCAT<br>ATGTGGCGGGACTCTTTATG	CACGCTGCTCAGTGTAGAGG<br>GAGGCGAGGATAACGAGCTA<br>GCGGCTCAACTCTCAAACTT	8.6<br>1.3<br>0.9
PPP2R1B	NM_002716	P1(-388/-194)<br>P2(+62/+200)	AAGAGGTGGAGGGGTTCTGT<br>AGCAGCAGGAGGAGAAAGAA	GAAGGAGCCAGGAACCTACC<br>TACCTGCACGTCTTCATTGC	1.9<br>2.2
PPP2R5E	NM_006246	P1(-556/-380)<br>P2(-198/-4)<br>P3(+48/+193)	AAAACAGGCACTGGGACATC<br>TTTTAGCCGGAAAGGATTCA<br>GAGAGAGAAAGGGGCATTGA	CAATCCCGGTGATCAATTTC<br>AATTTGGTCACGATGCACCT<br>GAAGGGAGGGAGGAAGACTG	2.1<br>3.4<br>1.4
Egr1	NM_001964	P1(-801/-643)<br>P2(-431/-317)	GAAAGACACCGTGCCATAGAT<br>GCCATATAAGGAGCAGGAAGG	GGTTCTATCGCTGTCATCCAG<br>CTTCTTCCCTCCTCCCAGAG	1.2<br>1.6
TRAIL	NM_003810	P1(-619/427)<br>P2(-201/-64)	TGTCCAGCCTAACACACAGG<br>AAATGGGCTTGAGGTGAGTG	AGGGTGGGAGTGGATAAAGG<br>GTCCCTCCCCTTTCCTACTG	1.3<br>1.5
GADD45A	NM_001924	P1(-580/-409)<br>P2(-296/-125)	AAATCAAACCAGCCATCAGG<br>GCCACCTCTAGCCTCTGCT	TCCAGGGTTAATGCACTGAAG<br>GCTCCTGGAGTCAGGTTGAG	1.7<br>1.8
FOXO3a	NM_201559	P1(-686/-516)<br>P2(-375/-274)	CAAACCTTTTGGTGCCTGAT<br>TCCCTCCTCTCCCCACTT	GTGTCCGGTTCCCTGTTAGA<br>CTCCCGAGAGTCTCAACGAC	1.2<br>1.1
CAV1	NM_001753	P1(-582/-413)<br>P2(-192/-90)	TGGCATAACCTGTTGGCATA<br>GGCAGGATTGTGGATTGTTT	GTCGGTGTGGATGGAAACTT<br>TTTTCGCAGTGCATCATCTC	0.9<br>1.3
TP53	NM_000546	P1(-175/-52)<br>P2(+269/+407)	GCAGGATTCCTCCAAAATGA<br>TCTCGGCTCCGTGTATTTTC	GAGGGTGCAGAGTCAGGATT<br>AGGTCTCCCAACAATGCAAC	1.4<br>2.1
